# Molecular Cloning, Characterization, and Application of Organic Solvent-Stable and Detergent-Compatible Thermostable Alkaline Protease from *Geobacillus thermoglucosidasius* SKF4

**DOI:** 10.4014/jmb.2306.06050

**Published:** 2023-11-15

**Authors:** Suleiman D Allison, Nur AdeelaYasid, Fairolniza Mohd Shariff, Nor'Aini Abdul Rahman

**Affiliations:** 1 Department of Bioprocess Technology, Faculty of Biotechnology and Biomolecular Sciences, Universiti Putra, Malaysia, 43400 Serdang Selangor, Malaysia; 2 Department of Biochemistry, Faculty of Biotechnology and Biomolecular Sciences, Universiti Putra, Malaysia, 43400 Serdang Selangor, Malaysia; 3 Department of Microbiology, Faculty of Biotechnology and Biomolecular Sciences, Universiti Putra Malaysia, 43400 Serdang Selangor, Malaysia; 4 Department of Food Science and Technology, Faculty of Agriculture and Agricultural Technology, Moddibo Adama University, Yola 640230, Nigeria

**Keywords:** Expression, thermostable, *Geobacillus thermoglucosidasius*, cloning, characterization

## Abstract

Several thermostable proteases have been identified, yet only a handful have undergone the processes of cloning, comprehensive characterization, and full exploitation in various industrial applications. Our primary aim in this study was to clone a thermostable alkaline protease from a thermophilic bacterium and assess its potential for use in various industries. The research involved the amplification of the SpSKF4 protease gene, a thermostable alkaline serine protease obtained from the *Geobacillus thermoglucosidasius* SKF4 bacterium through polymerase chain reaction (PCR). The purified recombinant SpSKF4 protease was characterized, followed by evaluation of its possible industrial applications. The analysis of the gene sequence revealed an open reading frame (ORF) consisting of 1,206 bp, coding for a protein containing 401 amino acids. The cloned gene was expressed in *Escherichia coli*. The molecular weight of the enzyme was measured at 28 kDa using sodium dodecyl sulfate polyacrylamide gel electrophoresis (SDS-PAGE). The partially purified enzyme has its highest activity at a pH of 10 and a temperature of 80°C. In addition, the enzyme showed a half-life of 15 h at 80°C, and there was a 60% increase in its activity at 10 mM Ca^2+^ concentration. The activity of the protease was completely inhibited (100%) by phenylmethylsulfonyl fluoride (PMSF); however, the addition of sodium dodecyl sulfate (SDS) resulted in a 20% increase in activity. The enzyme was also stable in various organic solvents and in certain commercial detergents. Furthermore, the enzyme exhibited strong potential for industrial use, particularly as a detergent additive and for facilitating the recovery of silver from X-ray film.

## Introduction

Enzymes are highly effective, environmentally benign protein catalysts that are produced by living organisms. Their advantages over chemical catalysts include specificity, high catalytic activity, the ability to operate at both moderate and high temperatures, and the potential for high yield [1]. Protease enzyme catalyzes the breakdown of protein molecules into simpler units, such as amino acids and peptides. Proteases are divided into four categories based on the functional group present in the active site. These include serine proteases, aspartic proteases, cysteine proteases, and metalloproteases [2, 3]. The pH at which serine proteases are optimally active is in the range of 7 to 11 [4]. The largest subgroup of serine proteases is serine alkaline proteases, which are active at extremely alkaline pH [1]. Thermostable enzymes are the most exploited and commercialized enzyme group, and as a result, they have strong industrial and varied research applications in diverse industries, including detergent, food, pharmaceuticals, leather, diagnostics, peptide synthesis, waste management, silver recovery, and food and beverage. Through these applications, thermostable enzymes are able to produce exceptionally high end-product yields [5, 6]. Approximately 40% of all enzyme sales worldwide are proteases derived from microorganisms [7]. The chosen bacteria must be capable of producing significant yields, secreting enormous amounts of protein, and being free of toxins and other unwanted chemicals while operating at high temperatures. The detergent and leather industries are only two examples of the many industries that frequently use thermostable alkaline proteases. However, their potential for usage in food and other applications, such as silver recovery from X-ray and photographic films, has not yet been completely investigated [3].

Proteases have been isolated from animal, plant, and microbial origins. The latter, however, is more often used since microbial proteases are resistant to changes in pH and temperature, as well as to conditions brought on by detergents and organic solvents [8]. One of the principal producers of microbial proteases, the *Geobacillus* species, has just been discovered. Examples include *G. stearothermophilus* F1 [9], *Geobacillus* spp. PA-5 and PA-9 [10], and *G. toebii* strain LBT 77 [11]. Accelerating protease synthesis in bacteria typically involves either recombinant DNA technology (rDNA) or conventional mutagenesis (UV or chemical exposure). For use in detergent formulations, proteases must be stable and active in difficult washing conditions of high temperature, alkaline pH, metal ions, and high salt concentration [1, 13, 14]. Although protein engineering and site-directed mutagenesis have been used to increase alkaline protease stability, screening microorganisms from harsh environments seems to be the most effective approach [11]. With the development of recombinant DNA technology and protein engineering, microorganisms can now be modified and expanded to meet rising demand [15]. Recombinant DNA techniques have allowed for the isolation and cloning of enzyme-encoding genes from all sources, including extremely challenging bacteria and other microorganisms [16, 17]. These techniques have also enabled the creation of high-yield heterologous proteins. The thermophilic, gram-positive, aerobic and facultative anaerobic bacilli that make up the genus *Geobacillus* thrive between 55 and 65°C [18]. Despite the fact that the genus has been discovered in different temperate climate regions, strains are frequently isolated from high-heat environments, including desert soil, compost, oil wells, and hot springs [19]. On the other hand, these species have long been prized as sources of thermostable proteins that act as strong biomimetic structures and stable catalysts [20, 21]. Another technique for extracting enzymes with improved thermostability involves isolating them from naturally occurring thermophilic species [22]. The optimum method, however, is to use recombinant DNA technology to clone and express the relevant thermophilic genes in mesophilic species [23]. As a foundation for manufacturing chemicals and fuel, the thermophile *G. thermoglucosidasius* has a lot of attraction [24]. This is the first report on the isolation of *G. thermoglucosidasius* from a hot spring, the cloning of an alkaline serine protease from the organism, and its usage as a detergent additive and in silver recovery from X-ray film.

At present, the synthesis of thermostable proteases by the available thermophilic bacteria is still insufficient. Therefore, much attention is paid to genetically modifying their enzymes to increase their activity, and to the screening of novel enzymes from new thermophilic bacteria sources to obtain the necessary properties, such as high stability in organic solvents and thermostability capacity for industrial and biotechnological applications [25, 26]. Due to their harsh growth conditions, it is difficult to grow the majority of the known thermophilic bacteria to make protease on a large scale [15]. The majority of thermostable protease enzymes continue to have functional and stability issues in heat and organic solvents [27]. The thermostable alkaline proteases now in use for industrial applications have certain drawbacks, including a deficiency in enzyme activity and stability with respect to contemporary bleach-based detergent formulations that comprise sodium dodecyl sulphate (SDS) and H_2_O_2_ [28, 29]. To solve these issues and limitations, we sought to isolate a more active and stable thermostable protease as previously reported from a highly thermophilic bacterium *G. thermoglucosidasius* from hot springs. The majority of industrial processes employ chemicals that are hazardous to the environment and are not environmentally friendly [30]. The scale of application for proteases is constrained despite their wide range of possible uses because there aren't any industrially significant functions among the existing proteases. As most industrial processes take place in hostile environments featuring harsh temperatures and pH levels, inhibitors, and other factors, proteases designed for industrial usage must be resistant to surfactants, oxidants, and be stable at high temperatures and pH [31-33]. In this study, we describe the cloning of a serine alkaline protease gene from *G. thermoglucosidasius*, its expression in mesophilic *E. coli*, purification, and characterization of the recombinant protease as well as its numerous applications.

## Material and Methods

### Strains, Plasmid, Media, and Culture Growth

The *G. thermoglucosidasius* SKF4 strain (Accession No. MN960021) was isolated in our previous work [34]. The competent host cells, *E. coli* BL 21(DE3), were used in the expression study. The primer used for PCR was obtained from Apical Scientific Malaysia. TransStart Fast PFU DNA Polymerase, DNA ligase, DNA molecular size marker, all restriction enzymes, gel extraction kit, PCR, and plasmid purification kit were all purchased from Beijing Transgen Biotech Co. *G. thermoglucosidasius* SKF4 was cultivated overnight in 10 ml Luria Bertani (LB) broth at 37°C and 200 rpm to obtain the inoculum culture.

The competent host cells (*E. coli* BL 21 (DE3)) were produced chemically using CaCl_2_ and stored in a freezer at -80°C for later use. (To obtain inoculum, the pEASY-Blunt E1 vector was inoculated into 10 ml LB broth containing ampicillin (10 mg) and incubated overnight at 37°C at 200 rpm. Two milliliters of inoculum was then inoculated into 100 ml LB broth containing ampicillin and incubated overnight at 37°C. The plasmid was extracted and prepared using plasmid DNA purification kits (Beijing Transgen Biotech Co., Ltd., China) according to the manufacturer's instructions.

### Primer Design and PCR Amplification of Thermophilic Serine Protease Gene

To obtain the complete nucleotide sequence of the thermostable serine protease gene, a pair of interspecific primers, SpSKF4-F and SpSKF4-R, was designed from the conserved regions around the nucleotide coding sequences (upstream and downstream) of the complete thermostable proteases genes of the following: *G. stearothermophillus* (Accession No. AY028615), *G. parathermoglucosidasius* (Accession No. NZ_LXMA01000010), and *Bacillus* sp. (Accession No. L29506.1). SpSKF4-F and SpSKF4-R have the following sequences: 5’-ATGAAGTTTAAAGCGATTGTTAG-3’ and, 5’-TTAATATGTTACAGCATTATAAGAATTG-3’, respectively. These conserved regions are amino acid sequences which are similar at the beginning of the sequences and the terminal end. The primers were used to amplify a fragment of the thermostable serine protease gene from *G. thermoglucosidasius* SKF4 in a 50 μl reaction mixture containing 2 ng of genomic DNA, 0.22 μM of forward and reverse interspecific primer, 25 μl of PCR master mix (Thermos Scientific), and 19 μl of deionized water. The PCR was incubated at an annealing temperature of 94°C for 4 min, followed by a later extension of 35 cycles of 94°C for 1 min, 45°C for 30 s, and a final extension at 72°C for 10 min. The amplified DNA product together with a DNA marker (100 bp ladder plus) was separated by electrophoresis using 0.8% agarose gel. The PCR products were gel-purified using a Gene All Purification Kit (GeneAll Biotechnology Co., Ltd., China) and sequenced by Apical Scientific Sdn Bhd (Malaysia).

### Digestion of Vector and Gene Insert with Restriction Enzymes

Digestion of the plasmid vector *pEASY*-Blunt E1 and gene insert SpSKF4 was done using appropriate Fast Digest restriction enzymes (Nde1 and Sac1) in the presence of a compatible buffer 10X Fast Digest buffer (Thermo Scientific Fast Digest, USA) at 37°C for 30 min. The digested product was checked with 12% agarose gel.

### Construction of the Recombinant Vector and Cloning of Thermostable Serine Protease Gene into a Linearized pEASY-Blunt E1 Expression Vector

The expression construct was prepared by ligation of purified serine protease and the purified linear pEASY-Blunt E1 cloning vector. The ligation mixture was properly mixed and incubated for 15 min at room temperature, before being placed on ice for 10 min. The purified construct was transformed into *E. coli* BL21 (DE3) cells using the CaCL2 heat-shock method [35]. Approximately 200 ml of transformants were spread on a selective agar plate containing IPTG (0.5 mM), X-gal (80 g/ml), and ampicillin (100 mg/ml) and incubated overnight at 37°C. The recombinant plasmids were confirmed by restriction digest using Fast Digest (Sac1 and Nhe1) restriction enzymes.

### Analysis of Sequence

All genes and proteins were analyzed using the BLAST search program (http://www.ncbi.nlh.nih.gov/blast). ClustalW version 3.2 was used to perform multiple sequence alignments of the serine proteases and their coding genes. The nucleotide signal peptide analysis was accompanied using a signal peptide prediction server (htpp://www.cbs.dtu.dk/services/Signal1P-3.0).

### Construction of Expression Plasmid

The forward and reverse interspecific primers without restriction sites were used to produce blunt-ended PCR products which were cloned directly to the linearized pEASY-Blunt E1 expression vector. The C-terminal His-Taq sequence of pEASY-Blunt E1 is followed by a linearized cloning site. The Nde1/Sac1-digested pEASY-Blunt E1gene fragments were introduced into the BL21 (DE3) expression host using heat shock transformation of *E. coli* BL21(DE3) competent cells prepared using the CaCL2 method at 42°C for 1 min [35].

### Expression of Thermostable Serine Protease SpSKF4 in *E. coli*

Chemically competent cells of *Escherichia coli* BL21 (DE3) were transformed with 2 μl of recombinant *pEASYS*pSKF4 construct in separate 1.5-ml tubes. Successfully transformed colonies of *E. coli* BL21 (DE3) were randomly selected on LB agar plates supplemented with 100 μg/ml of ampicillin after incubation for 18 h at 37°C. A 10 ml LB broth was used to grow a single colony of transformed *E. coli* BL21 (DE3) supplemented with the same antibiotic concentration in a universal bottle at 37°C and 200 ×*g* for 18 h. Fifty-milliliter cultures were incubated at 37°C and then shaken at 200 rpm until the OD reached 0.6. Following that, IPTG was added to a final concentration of 0.4 mM, and the cells were cultured for 8, 12, 18, and 24 h at 20°C. The cultures were then harvested at 8,000 ×*g* for 15 min. The supernatants were discarded, and the pellets were kept at -80°C until needed.

### Western Blot Analysis of Serine Protease Protein

The western blot was performed according to the manufacturer’s instructions. The ice-thawed pellets were suspended in 10 ml (20 mM sulphate buffer) at pH 7.5, containing 0.5 mM NaCl, and lysed by sonication. The lysates were centrifuged at 8,000 ×*g* for 15 min at 4°C. The pellets and the supernatants were assayed using sodium dodecyl sulphate polyacrylamide gel electrophoresis (SDS-PAGE) on 12% gel. Then, 9 μl of insoluble and soluble fractions were mixed with 1 μl of 10x sample buffer and electrophoresed on 12% gel. To carry out the western blot analysis, protein bands on the gel were transferred into a nitrocellulose membrane using a trans-Blot SD Semi-Dry Electrophoresis Transfer cell (Bio-Rad, Germany) at a constant current of 0.45 mA for 1 h. Blotting and detection of the presence of histidine tag in recombinant protein were done using a Western Breeze Chromogenic Western Blot Immunodetection Kit (Invitrogen, USA). Primary antibody (IgG Mouse) and (IgG anti-mouse alkaline phosphate) and PVDF membrane were used for detection.

### Purification of Recombinant Serine Protease SpSKF4

The recombinant *pEASY*-Blunt E1/SpSKF4 was cultured at 20°C for 12 h in a culture volume of 200 ml containing 0.4 mM IPTG. IPTG was added when the OD reached 0.6. The soluble proteins were subjected to purification processes. Purification of the His-tagged recombinant protease was carried out by utilizing a one-step Ni-Sepharose affinity chromatography process. A Ni-Sepharose 6 Fast Flow column (XK16/20) was loaded with filtered recombinant crude extract (GE, USA). Binding buffer (50 mM Tris-HCl, 5 mM imidazole, 500 mM NaCl, pH 8) was used to equilibrate the column at a flow rate of 5 ml/min. The crude protein was fed onto the column, and the bound protein was eluted using an isocratic elution buffer of 50 mM Tris-HCl, 500 mM imidazole, and 500 mM NaCl, pH 8 [29]. Protease assay, SDS-PAGE, and native PAGE were used to confirm that eluted fractions contained the protein of interest [36].

### Sodium Dodecyl Sulphate Polyacrylamide Gel Electrophoresis (SDS-PAGE)

Pellets were thawed on ice and suspended in 10 ml sulphate buffer 20 mM (pH7.5) containing 0.5 mM NaCl and lysed by sonication. The lysate was centrifuged at 8,000 ×*g* for 15 min at 4°C. Both the pellets and supernatants were assayed by sodium dodecyl sulphate polyacrylamide gel electrophoresis (SDS-PAGE) on 12% gel [37](Laemmli, 1970). Nine microliters (9 μl) of either the supernatants or the pellets were mixed with 1 μl of 10x sample buffer and loaded onto the gel. Gel electrophoresis was performed using 1X Tris-glycine buffer at 3% (w/v) Tris,14.4 % (w/v) glycine, 0.1% (w/v) SDS, pH 8.4) at constant current of 60 mA and 210 V for 45 min. Coomassie Brilliant Blue R-250 (0.5% w/v) in 25% (v/v) isopropanol and 10% (v/v) acetic acid were used to stain the gels which were allowed to stand at room temperature for 10 min under gentle shaking. The gels were immediately destained with a solution that contained 10% methanol in water (v/v) and 10% (v/v) acetic acid for several hours until the recombinant proteins were visible.

### Characterization of Serine Alkaline Protease

**Protease Activity Assay**

The protease activity was assessed by a modified method described by MacDonald and Chen [38] using casein as substrate. This method involved the use of three sets of test tubes, two experimental and one for control. In each of the three test tubes, 2 ml of 1% casein in Glycine-NaOH buffer pH 10 was added. One milliliter of the enzyme was added to two experimental tubes, each containing 2 ml of 1% casein. The three test tubes were incubated at 60°C for 30 min. The reaction was stopped by adding 3 ml 10% TCA and allowed to cool down for 10 min at 4°C. The reaction mixtures were centrifuged at 12,000 ×*g* for 10 min. Then, 1 ml of the supernatant was added to 5 ml Na_2_CO_3_. Half a milliliter of Folin-Ciocalteau reagent (diluted at 1:1) in distilled water was added in each test tube and incubated for 30 min. At the end of the incubation, absorbance was read using a spectrophotometer at 700 nm. Under the above assay conditions, one unit of enzyme activity is defined as the amount of enzyme that releases tyrosine at a rate of 1 g/min [39, 40].

### Determination of Protein Concentration

The Bradford method was used to determine the total protein concentration of a sample, with bovine serum albumin (BSA, 0.2 mg/ml) as the standard [41] (Bradford, 1976). The standard calibration curve was created in response to BSA absorbance values made in various concentrations. The standard calibration curve equation was used to quantify total protein content. As described by the Bradford method, the Coomassie Brilliant Blue G-250 dye binds to arginine, lysine, and histidine residues in proteins and alters their color. The change in the absorbance was determined spectrophotometrically at 595 nm, using a UV-visible spectrophotometer.

Protein concentration (mg/ml) = Absorbance value/Gradient.

### Specific Activity Determination of Serine Protease

The specific activity of an enzyme is the amount of product generated by the enzyme in a particular amount of time under specific conditions [31]. The formula below was used to calculate the specific activity of the serine alkaline protease. By dividing the enzyme activity (Units) by the protein content (mg) and expressing the result as U/mg protein, the specific activity can be determined.

$$\text{Specific activity (U/mg) = }\frac{\text{Enzyme activity (U/ml)}}{\text{Protein (mg/ml)}}$$

### Effect of pH and Temperature on the Activity and Stability of Alkaline Protease

The effect of temperature on the activity and stability of the alkaline protease was performed according to Rekik *et al*. 2019 [42]. The optimum temperature for protease activity was determined over the range of 20–100°C by the protease assay. Assay mixtures were equilibrated at the required temperature before adding the enzyme. The effect of temperature on protease stability was determined by incubating aliquots of the purified enzyme for 24 h in 0.1 M Glycine-NaOH buffer (pH 10) at temperatures of 80, 85, 90, and 95°C. The non-heated enzyme, cooled on ice, was considered as control (100%). The relative activity was then determined under standard assay conditions. Protease activity was measured at 80°C over a pH range of 2–13. The optimum activity was determined at 80°C using the following buffer systems (100 mM each): Hydrochloric acid/potassium chloride buffer (pH 1-2), citric acid/sodium citrate buffer (pH 3-5), phosphate buffer (pH 6-7), Tris amino methane/hydrochloric acid buffer (pH 8-9), sodium Glycine/sodium hydroxide buffer (pH 10), and sodium phosphate dibasic/sodium hydroxide (pH 11-12) the highlited phrase deleted [43]. The pH stability of SpSKF4 was tested by pre-incubating the enzyme for 1 h at the optimum temperature of 80°C in buffer solutions with various pH values, and the residual activity (pH 10 optimum) was evaluated. Enzymatic activities were assessed using the previously described standard conditions [38]. The activity and relative activity of the protease enzyme were estimated.

### Effect of Various Metal Ions on Protease Activity

The effect of various metals was determined using the method of Thebti *et al*. [11] with modification. To determine the effect of various metal ions on the protease activity, the enzyme was incubated with 1 mM of different metal ions solution for 30 min, and the protease activity was later measured as explained in Section 3.3.3. The metal ions that were evaluated included K^+^ Ca^2+^, Na^+^, Mg^2+^ F^2+^, Mn^2+^, Ni^2+^, Zn^2+^, and Cu^2+^, which were contributed by metal salts, namely calcium chloride, sodium chloride, magnesium sulphate, ferric chloride, manganese chloride, nickel chloride, zinc sulphate, copper sulphate, and aluminum sulphate, respectively. The enzyme and metal ions were pre-incubated for 10 min at 80°C before buffer and substrate were added and incubated for 30 min at 80°C and pH 10. The residual activity was estimated, and the enzyme activity of the control (in the absence of metal ions) was assumed to be 100%. The activity of the enzyme and residual activity were estimated.

### Effect of Organic Solvents on Protease Activity

The influence of solvents such as acetone, ethanol, isopropanol, methanol, hexane, chloroform, and propanol on the activity of serine alkaline protease was investigated using the method described by [44] with modifications. This was done by incubating the enzyme with each solvent at concentrations of 15, 25, and 50% for 10 min at 80°C, and then incubating for 30 min at 80°C before performing the protease assay as reported before in *Protease Activity Assay*. The enzyme activity of the control (without solvents) was taken as 100% and the relative activity was calculated.

### Effect of Surfactants on Protease Activity

The effect of surfactants (Tween 20, Triton 100, and sodium dodecyl sulphate) with varying concentrations of 5 and 10 mM on the stability of alkaline protease was investigated by pre-incubating the enzyme with each surfactant for 10 min at 80°C before performing the protease assay as described previously. The residual activity was calculated with the enzyme activity of the control (without surfactants) being assumed to be 100% [31].

### Commercial Detergent Compatibility Studies

The enzyme stability in commercial detergents, which were obtained from Giant Shopping Mall, South City Malaysia, was carried out using different detergents, namely Freeze, Top, Brezee, Fab perfect, Bio Zip, and Depex at the concentration of 5 mg/ml. The experiment was carried out according to Suberu *et al*. [31] with few modifications. The detergents were heated in boiling water for 10 min to inactivate the enzyme present in the detergents. The protease enzyme solution was mixed with the solution of the detergents for 1 h at 80°C and the protease enzyme activity assay was performed.

### Effect of Inhibitors on Protease Activity

The effect of inhibitors was performed according to the method of Thebti *et al*. [11] with modifications. After incubation with various concentrations (5, 10 mM) of phenyl methane sulfonyl fluoride (PMSF), ethylene diamine tetra acetic acid (EDTA), aprotinin, and iodoacetamide at 80°C for 1 h, the activity of the purified protease was measured. The relative activity was calculated spectrophotometrically, following a protease enzyme assay under standard conditions as described in Protease Activity Assay. The enzyme activity of the control (without inhibitors) was taken as 100%.

### Effect of Oxidizing Agents on Protease Activity

The effect of oxidizing agents was performed according to Thebti *et al*. [11] with modification. To investigate the effect of oxidizing agents such as dimethyl sulfoxide (DMSO) and hydrogen peroxide on the activity of protease enzyme, the enzyme was pre-incubated with each oxidizing agent at a concentration of 1-5% (v/v) for 10 min at 80°C, and then incubated at 80°C for 30 min before performing the protease assay as described previously.

### Effect of β-Mercaptoethanol as a Reducing Agent on Protease Activity

To investigate the effect of β-mercaptoethanol on the activity of the enzyme, the enzyme was pre-incubated with β-mercaptoethanol at a concentration of 25 and 50% (v/v) for 10 min at 80°C, and then incubated at 80°C for 30 min before performing the protease assay as described previously. The enzyme activity of the control (without β-mercaptoethanol) is assumed to be 100% [31].

### Determination of Substrate Specificity of the Protease

The substrate specificity of the enzyme was determined using the method of Yildirim *et al*. [36]. Casein, azo-casein, haemoglobin, bovine serum albumin (BSA), keratin, and gelatin substrates were used to assess the substrate specificity of the SpSKF4 protease enzyme. Glycine-NaOH buffer (pH 10) was used to prepare 1% of each substrate at the optimum temperature of 80°C. After that, the activity of 0.5 ml of enzyme and 2.5 ml of substrate solution was tested. The relative enzyme activities were determined using the enzyme's activity and the substrate which demonstrated the most activity as described previously.

### Nucleotide and Protein Sequence Accession Numbers

*Geobacillus* sp. strain SpSKF4 (34) and its 16S rRNA nucleotide gene sequences were deposited in the NCBI GenBank database under Accession No. MN960021, and the SpSKF4 thermostable serine protease gene nucleotide and protein sequences were deposited in the NCBI GenBank database under Accession No. MZ041100.

### Applications of Thermostable Serine Protease

Various potential and biotechnological evaluations of the purified recombinant SpSKF4 enzyme were carried out to determine its industrial applications and washing capacity, particularly in detergent.

### Wash Performance Studies

To assess the effect of protease on stain removal, water was substituted with buffer (50 mM Glycine NaOH, pH 10.0). Visualization was used to check the capacity for stain removal. To assess the wash performance of the partially purified protease, a piece of white cotton cloth (1.5 cm × 1.5 cm) was stained with red blood. The red blood-stained cloth strips were sun-dried for 12 h and then placed in 250-ml Erlenmeyer flasks labeled A-D before being subjected to a temperature of 80°C at pH 10 in 100 ml of the reaction mixture under different sets. The following wash treatment was performed according to Corrêa, *et al*. [45].

Conical flask A contained 100 ml distilled water + piece of red blood-stained cloth; Conical flask B contained 100 ml detergent solution + piece of red blood-stained cloth; Conical flask C contains 100 ml detergent solution + piece of red blood-stained cloth + 1 ml partially purified enzyme sample of SpSKF4 protease; Conical flask D contained 100 ml detergent solution + piece of red blood-stained cloth + 1 ml partially purified enzyme SpSKF4 protease and Conical flask E: 100 ml + piece of red blood-stained cloth + 1 ml of partially purified protease from *Bacillus licheniformis* 2D55 as a positive control. The mixtures were incubated at 80°C for 30 min in a water bath (MRC). After incubation the cloth pieces were removed and rinsed in tap water, and dried. The effectiveness of the removal of the blood stains was determined by visual examination. As a negative control, untreated pieces of white cloth stained with blood were used [46].

### Decomposition of Gelatin Layer of X-Ray Photographic Film

The decomposition of the gelatin layer for the recovery of silver from X-ray films was performed according to Patil *et al*. [47] with a few modifications. The capability of the protease enzyme in decomposing layer of gelatin in X-ray film was carried out with incubation of 2 g of X-ray film (1 cm × 1 cm) in 1 ml solution of 350 U/ml enzymes. The experiment was set up as follows:

Flask 1 contained 20 ml Glycine –NaOH buffer + 2 g of X-ray film + 1 ml enzyme sample (SpSKF4). Flask 2 contained 20 ml Glycine-NaOH buffer + 2 g of X-ray film + 1 ml enzyme (*B. licheniformis* 2D 55 enzyme) positive control, and Flask 3 contained 20 ml Glycine-NaOH buffer pH10 only (negative control). The flasks with the mixtures were kept in a rotary shaker (Scigenic Biotech, Country) at room temperature and 120 rpm for 3 h. The X-ray films were removed at every 1 h interval, washed in tap water, and a visual check was carried out. The amount of protein removed from the X-ray film into the solution by the enzyme was also calculated according to Bradford, *et al*. [41] at 1 h interval as described previously. The test was performed in triplicate.

### Statistical Analysis

All of the experiments were carried out three times, and the mean and SD were calculated using Microsoft Excel 2007 (Microsoft Corp., USA). Sigma plot for Windows 11.0 was used to create the graphs (Systat Software Inc., Germany).

## Results and Discussion

This research focused on cloning the serine alkaline protease gene from *Geobacillus thermoglucosidasius* SKF4, and the potential applications were also evaluated. In this study, a new gene named SpSKF4 was amplified and cloned from *G. thermoglucosidasius* SKF4 for the first time.

### Analysis of the Gene and Amino Acid Sequences

The amplified gene measures 1,206 bp and encodes a sequence of 401 amino acids, consistent precisely with the anticipated size of the gene. Subsequent successful expression of the SKF4 gene was achieved in the BL21 expression host. The products expressed demonstrated an approximate molecular weight of 28 kDa, a finding that was further confirmed through western blot analysis (Fig. 4A and 4B).

**Fig. 1:**
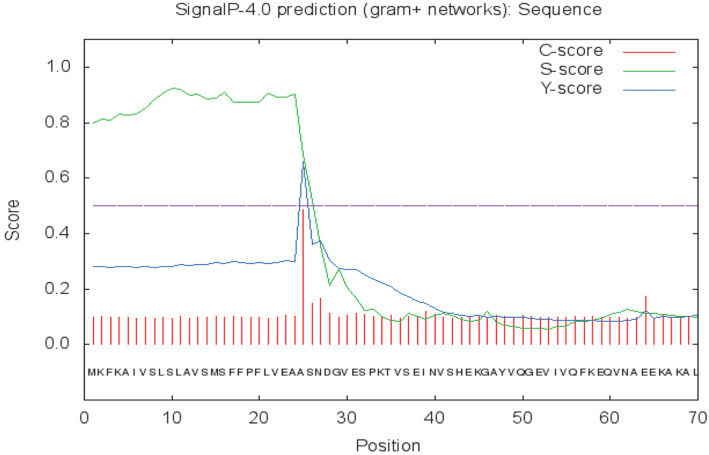
Signal peptide analysis of the predicted amino acid sequence of *G. thermoglucosidasius* SKF4 serine protease gene. C- score 0.6; S-score 0.8 and C-score 0.5.

The analysis of the SpSKF4 protease gene sequence revealed the presence of an open reading frame (ORF) responsible for encoding a potential serine protease precursor comprising 401 amino acid residues. Upon subjecting the deduced amino acid sequence to SignalP 4.0 analysis, a hydrophobic signal peptide was revealed to be located at the N-terminus. Notably, the cleavage site for the signal peptides sequence was found to be positioned between Ala25 and Ser26, contributing to a significant mean S value of 0.8 (Fig. 1).

This signal peptide plays a crucial role in both the targeting and translocation of proteins within prokaryotic and eukaryotic cells. The high S-score signifies indicates the potential of efficient protein translocation across the cell membrane [23].

**Fig. 2:**
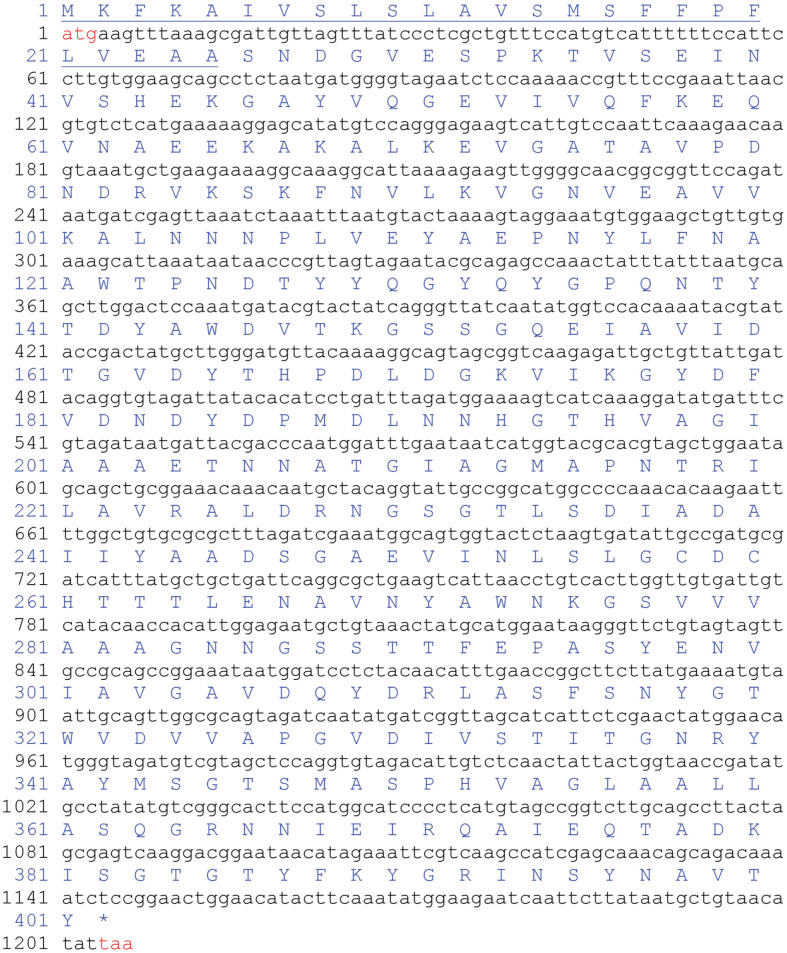
Complete nucleotide sequence of *G. thermoglucosidasius* SKF4 serine protease gene. The nucleotide bases sequences are shown in black small letters with start and stop codons in red (taa). Sequences of the amino acids are shown in Blue capital letters. The underlined sequences indicate the amino acid sequence of the signal peptide.

An analysis of the enzyme’s ORF gene sequence revealed that it begins with a 25-amino acid signal peptide, followed by a 97-amino acid propeptide, and a 279-amino acid mature polypeptide (Figs. 1 and 2). Using the Compute PI/MW tool, the putative SpSKF4 protein was estimated to have a theoretical molecular weight (MW) of 41.043 kDa and an isoelectric point (pI) of 4.50. The complete DNA sequence of SpSKF4 comprised 1,206 bp, starting with an initiation codon (ATG) at nucleotide position 1, and ending with a termination codon (TAA) at nucleotide position 1206 (Fig. 2).

**Fig. 3:**
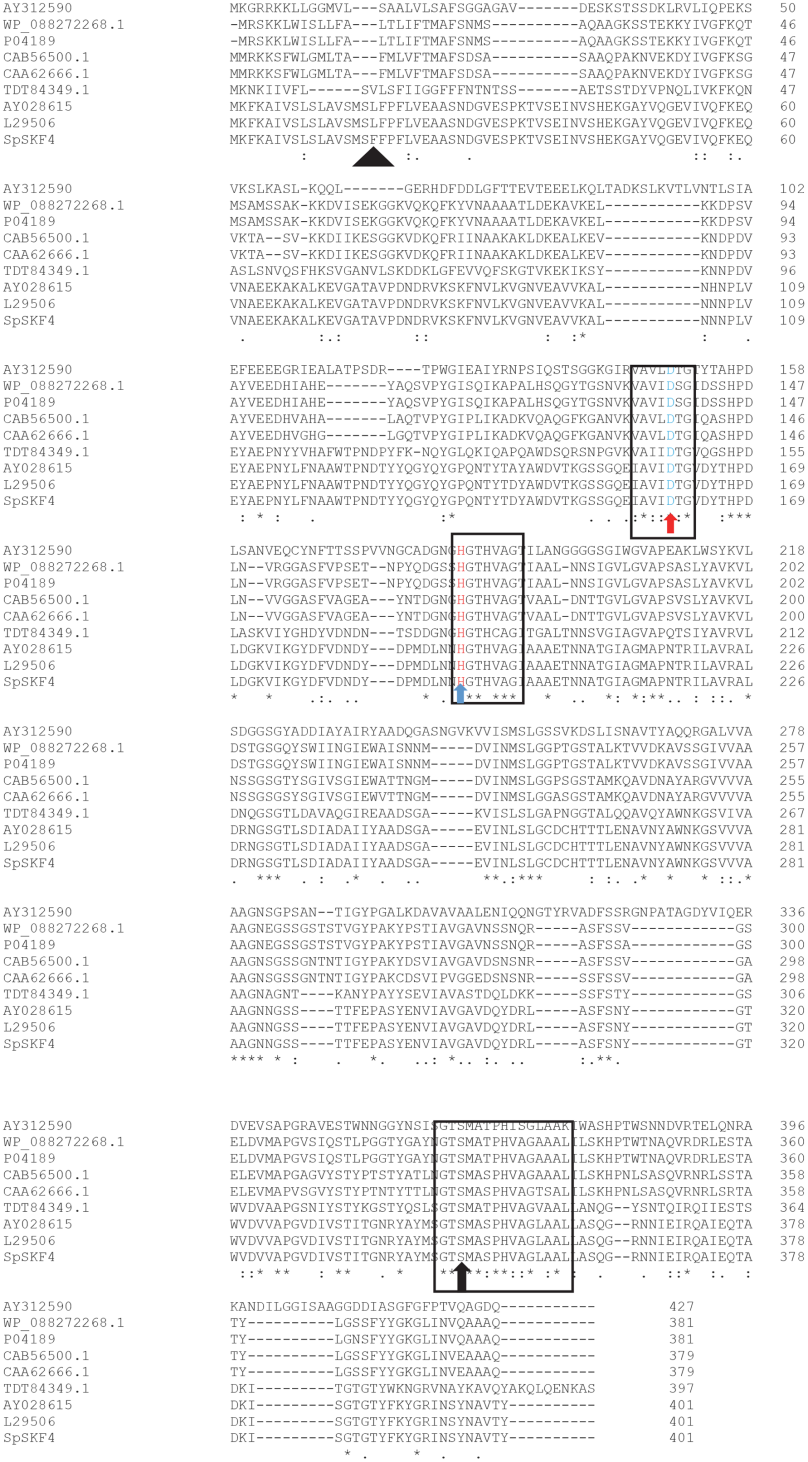
Multiple sequence alignment of the deduced amino acid sequence of SpSF4 with other proteases. Bacillus sp. WF146 protease subtilisin-like (AY312590), sp|P04189 Subtilisin E from *B. subtilis*, TDT84349.thermitase from *Bacillus* sp.(AG1163), WP_088272268 Subtilisin AprE from *B. subtilis*, CAA62666 Subtilisin Carlsberg from *B. licheniformis*, CAB56500 SubC Subtilisin BPN’ from *B. licheniformis*, L29506 Ak1 serine protease from *Bacillus* sp., AY028615 F1 protease from *Geobacillus stearothermophillus*, SpSkF4 serine protease from *G. thermoglucosidasius* SKF4 (this study). The black rectangle shows the area of high conserve region. The catalytic triad are shown by red arrow (Aspartic acid), blue arrow (Histidine) and black arrow (Serine). The black triangle shows the amino acid phenylalanine replacement in the signal peptide.

When the homology sequence of the ORF of the protein encoding 401 amino acid residues of serine protease of *G. thermoglucosidasius* SKF4 was compared to other subtilisin-like proteases, a high level of similarity (>70%) was revealed, along with a high number of conserved regions (Fig. 3).

The positions of aspartate, histidine, and serine in the complete sequence of SpKF4 protease amino acid were found at positions 160, 193, and 363, respectively, and these are conserved throughout the serine proteases shown in alignment (Fig. 3) [23, 48]. The sequence of the deduced amino acids of the SpSKF4 serine protease is similar in its characteristics to other signal peptides, which show two basic lysine residues and a high amount of hydrophobic amino acid sequence [23]. The gene sequence of the SpSKF4 gene displayed high homology with the family of subtilisin, which represents the major group of the category of serine proteases [23]. The study of both the gene and amino acid sequence revealed an OFR of 1,206 bp, which translates to a sequence encoding 401 amino acids. According to findings from the MEROPS peptidase database (http://merops.sanger.ac.uk), SpSKF4 is classified within the subtilisin-like protease family (S8A subfamily, clan SB) [23]. Particularly, the protease contains a highly conserved catalytic triad, composed of Asp160, His193, and Ser 363, crucial for its enzymatic activity [48]. This catalytic triad serves a dual role in stabilizing the oxyanion tetrahedral transition state and facilitating the secretion of the protein across the membrane [48, 49]. This information highlights the functional role of these residues in the mechanism of action of SpSKF4 protease.

The propeptide was found to function as an intramolecular chaperone (IMC), acting as a template for the mature domain of the protein and aiding its proper folding [49]. The presence of the signal peptide and prepropeptide domain at the N-terminal of the deduced amino acid sequence suggests that SpSKF4 was either synthesized or cloned as a preproenzyme, as observed in the study by Ekchaweng *et al*. [48].

Subtilisin-like proteases from species such as *Thermus aquatus* [50], protease Ak1 from *Bacillus* sp. [23](Maciver *et al*., 1994), protease F1 from *G. stearothermophilus* [9], and aqualisin I from *T. aquaticus* [51] are all known to have prosequences identified to play a significant role in activating the protease enzyme as intramolecular chaperons. The prosequences are removed from the catalytic domain of these proteases by autoprocessing during expression and can also be removed by other protease enzymes [50, 52].

The results of alignment of multiple amino acid sequences among the predicted ORF of some other proteases, such as Ak1 [23] F1 [9]), subtilisin AprE [53], and subtilisin BPN’ [54] showed high levels of similarity with a high number of the conserved regions (Fig. 3). However, the characterization and applications of these proteases, particularly in the areas of the present study, have not been fully investigated.

### Expression and Partial Purification of the *G. thermoglucosidasius* SKF4 Recombinant Protease Gene in *E. coli* BL21 (DE3)

**Fig. 4:**
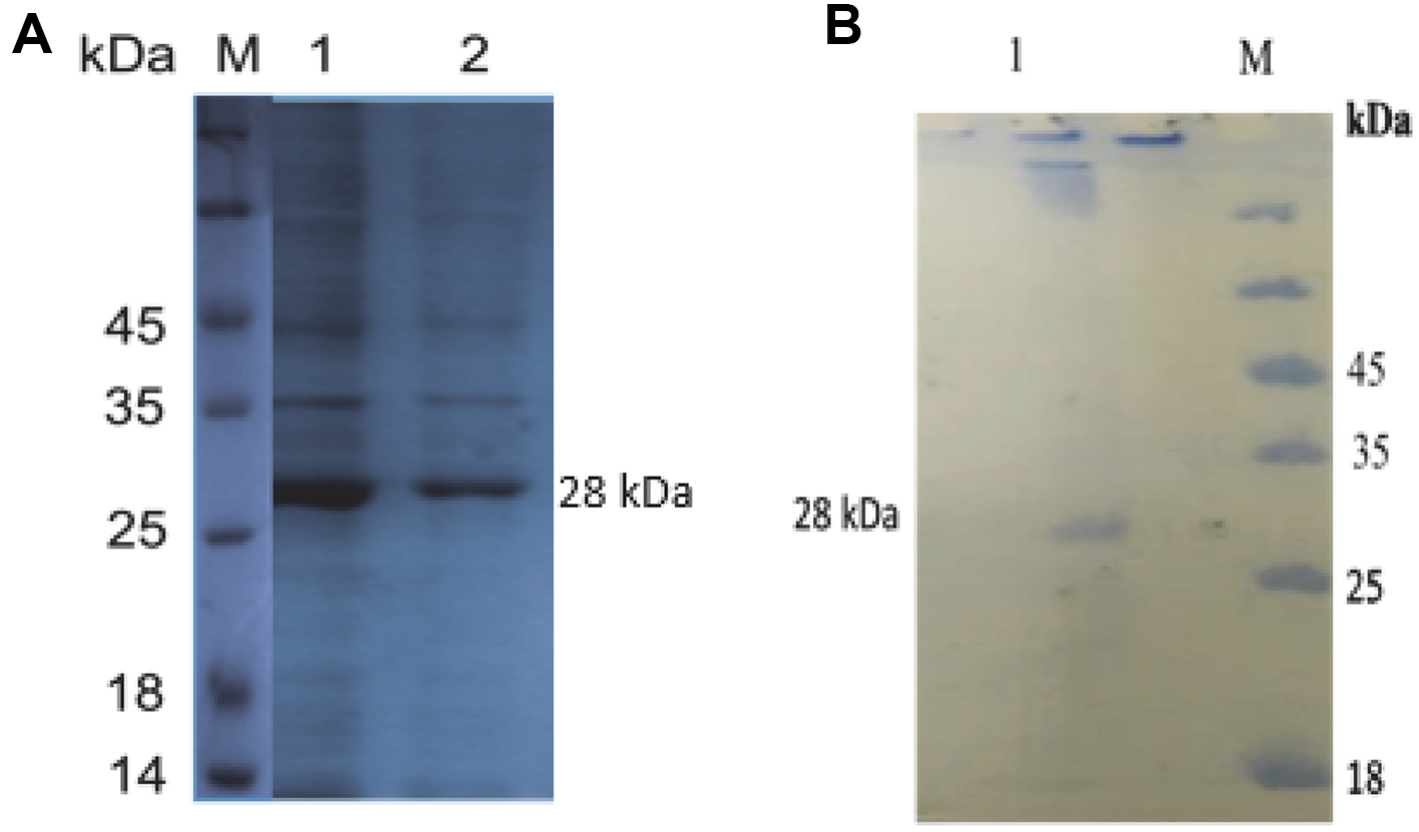
(A) Partial purification of recombinant SpSKF4 protease, (B) western blot analysis of SpSKF4 protease showing mol. wt of approx. 28 kDa.

The expression of the SpSKF4 protease gene in *E. coli* BL21 (DE3) was investigated by selecting positive clones of transformants. To determine and analyze the expression of the SpSKF4 protease gene, both SDS-PAGE and western blot techniques were employed. The results of these analyses, (Fig. 4A and 4B) respectively, displayed a single band of approximately 28 kDa corresponding to the molecular weight of the recombinant protease This observation confirms that the protein was expressed in its mature enzyme form [23].

The nucleotide and amino acid sequences of the SpSKF4 protease have been officially submitted and deposited in the GeneBank database under the Accession No. MZ041100. This ensures that the sequences are publicly accessible and can be utilized by the scientific community for future research and reference purposes. Previous research has actually explored the cloning of alkaline protease genes from various *Geobacillus* and *Bacillus* species [9, 23, 31, 55, 56]. However, in the current investigation, a new objective was achieved by cloning the SpSKF4 gene from *G. thermoglucosidasius* into the pEASY-Blunt E1 expression vector for the first time. In this investigation, SDS-PAGE was applied to validate the production and purification of the serine alkaline protease enzyme. The results of this analysis revealed that the molecular weight of the serine protease was approximately 28 kDa (Fig. 4A and 4B).

However, the estimated molecular weight of SpSKF4 was 41.3 kDa using the Expasy online tool (http://web.expasy.org/cgi-bin/compute_pi/pi_tool). This indicates there was an autoprocessing procedure during the expression which cleaved the prosequence and the signal peptide and hence the protein was expressed as a mature protein [23]. Various researchers have previously investigated the cloning, expression, and characterization of different serine alkaline proteases with varying molecular weights falling within the range of 18 to 45 kDa [31]. For instance, Suberu *et al*. (2019) recorded the expression of an alkaline protease from *Bacillus* sp. BR7, with a molecular weight of 43 kDa [9]. Also, Fu *et al*. (2003) reported the expression of an alkaline protease from *G. stearothermophilus* F1, which exhibited a molecular weight of 27 kDa [56].

Moreover, a cloned and characterized alkaline serine protease from *G. stearothermophilus*, with a molecular weight of 39 kDa, has been previously reported [56]. These results illustrate the richness and diversity within this specific enzyme category and show the wide range of molecular weights displayed by serine alkaline proteases. The results of the present study also agree with the findings of previous researchers who have reported that the MW of alkaline serine proteases falls within 15-30 kDa [57-60]. In addition, other researchers have reported the discovery of serine alkaline proteases with MWs as low as 8 kDa [61], while still others have documented higher MWs reaching up to 45 kDa [62] and 43 kDa [31]. However, the MW of the expressed and purified SpSKF4 protease falls within the range of these reported alkaline proteases. Additionally, 352 U was shown to be the ideal protease activity of the expressed serine protease enzyme (Table 1). This finding not only verifies that the cloned gene was overexpressed in its active form but also underlines the success of the cloning process, resulting in the production of substantial quantities of a specific protein exhibiting high enzyme activity. This successful expression and activity validation further highlight the practical usefulness of the cloned SpSKF4 gene in producing an effective and industrially relevant enzyme.

The purification table shows that purification by heat treatment has a protein recovery of 62% and a purification fold of 1.6, while the affinity chromatography using IMAC produced a protein recovery of 11% and a purification fold of 8.6 for the recombinant protease and a total protein of 2.5 mg (Table 1).

### Characterization of Partially Purified Recombinant SpSKF4 Serine Protease

**Effect of Temperature and pH on the Activity and Stability of the Purified Protease**

**Fig. 5:**
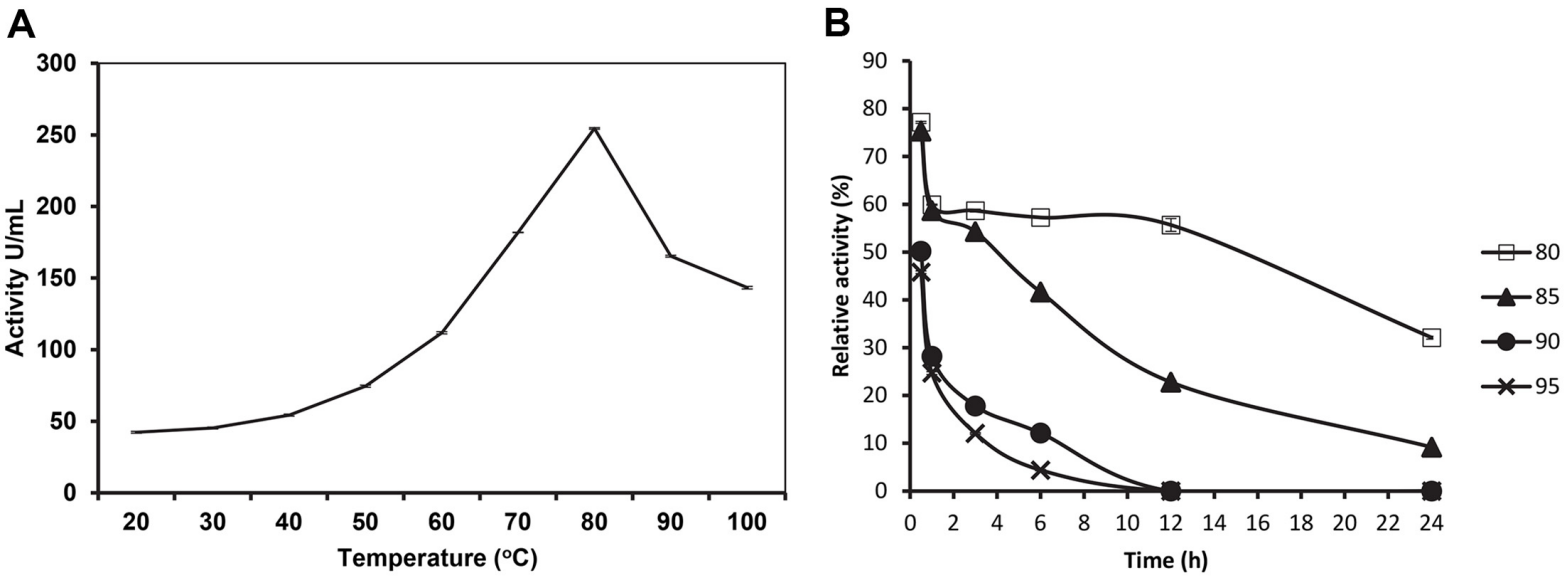
(A) Effect of temperature on the activity of purified SpSKF4 protease. (B) Temperature stability of SpSKF4 protease which show half-life at temperure of 80°C, 85°C, 89°C, and 95°C.

**Fig. 6:**
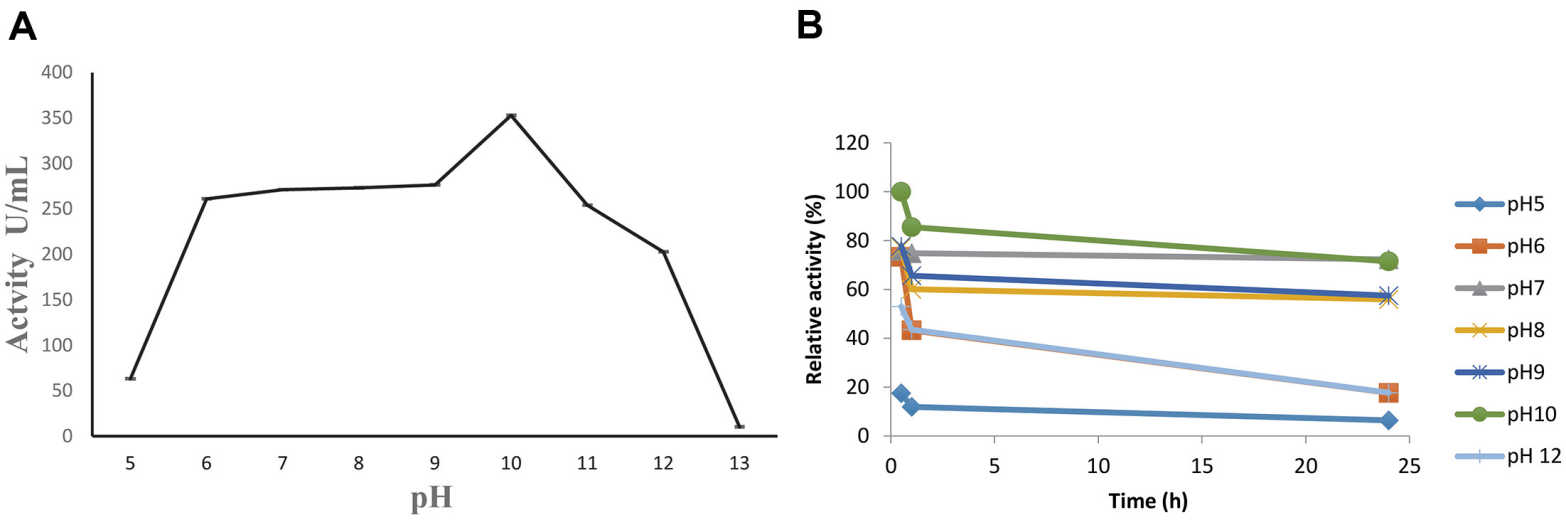
(A) Effect of pH on the activity of SpSKF4 protease. The substrate casein was produced in the appropriate pH buffer (pH 3-5), phosphate buffer (pH 6-7), Tris amino methane/hydrochloric acid buffer (pH 8-9), sodium Glycine/sodium hydroxide buffer (pH 10) and sodium phosphate dibasic/sodium hydroxide (pH 11-12) were the different buffer systems used (Vincent and John, 2009). (B) Effect of pH on stability of SpSKF4 protease at optimum temperature.

A variety of pH and temperature conditions were used to comprehensively evaluate the stability and activity of the SpSKF4 alkaline protease. The enzyme revealed outstanding stability at this temperature, with an estimated half-life of 15 h, and showed its maximal activity at a temperature of 80°C (Fig. 5B). Furthermore, the enzyme's optimal activity was observed at pH 10 (Fig. 6A), and it demonstrated remarkable stability under alkaline conditions for over 24 h (Fig. 6B). Furthermore, the enzyme's activity and specific activity were 352 U/ml and 141 u/mg of protein, respectively, confirming its distinctive alkaline and thermostable characteristics (Table 1). This comprehensive evaluation emphasizes the robust nature of the SpSKF4 alkaline protease, highlighting its potential applicability in various industrial and biotechnological processes.

These findings are consistent with previous studies that found that serine proteases from *Geobacillus* and *Bacillus* species are active at a broad range of pH and temperatures [63]. An optimum temperature of 85°C was recorded for a protease enzyme isolated from *G. stearothermophilus* in the presence of 2 mM calcium ion. However, they also reported optimum activity of F1 protease at 75°C in the presence of calcium ions. The enhanced stability and activity observed in the SpSKF4 alkaline protease could be attributed to its origin from *G. thermoglucosidasius* SKF4, which was isolated from a hot spring environment [63]. The results of the present investigation concerning the SpSKF4 protease are in good agreement with the findings of Zhu *et al*. [64], who determined the optimal activity of a protease enzyme isolated from *Geobacillus* sp. YMTC 1049 at 85°C. Both studies demonstrate that enzymes obtained from *Geobacillus* species can operate efficiently at high temperatures, in this case, in the range of 80-85°C. This shows that SpSKF4 is a highly thermostable protease. The protease enzyme SpSKF4 has a broad range of activity from 40-100°C (Fig. 5A). This result also agrees with Zhu *et al*. [64] who showed a similar broad activity at high temperatures of (60-100°C). The broad temperature activity of 40-100°C indicated the capability of the enzyme SpSKF4 for both biotechnological and industrial applications.

According to Fig. 5B, the enzyme had a half-life of 1 h at 90°C and continued to be active for 6 h. The enzyme's half-life was also 1 h at 95°C, and it increased to roughly 4.5 h at 85°C (Fig. 5B). The enzyme maintained 77% of its activity after 30 min and 60% after 4 h at its optimum temperature of 80°C. With a half-life of roughly 15 h, the enzyme was still active after 24 h of incubation at the ideal temperature of 80°C (Fig. 5B). However, its activity was only about 40% of what it had been initially (Fig. 5B).

Numerous studies have shown that enzyme activity decreases over time and at higher temperatures. For instance, the Ak1 protease has a half-life of 1.7 h at 85°C and a staggering 12.4 h at 80°C [23]. This level of stability is significantly higher than that of *G. stearothermophilus* F1 protease, which has a half-life of 3.5 h at 85°C and just 25 min at 90°C [9]. The SpSKF4 protease has a half-life of 1 hour at 90°C and increases to about 4.5 h at 85°C. This demonstrates SpSKF4 protease's superior thermostability when compared to other reported proteases.

According to this study, the SpSKF4 protease has the potential for useful industrial applications because of its capacity to remain active over a wide temperature range of 20–100°C. Numerous factors, some of which have been discussed by earlier researchers, contribute to the stability of thermostable enzymes like SpSKF4. For instance, it has been hypothesized that calcium supports β-amylase's thermostability by keeping its native conformation, which offers the requisite structural stability required for efficient catalytic activity at high temperatures [70, 71].

The serine protease enzyme demonstrated activity in a pH range of 5-12, with the highest protease activity at pH 10, indicating that the recombinant protease SpSKF4 is characteristically alkaline. Several reports have revealed the production of thermostable alkaline proteases derived from various strains of *Bacillus* and *Geobacillus*. Fu, *et al*. [9] reported that broad activity at pH 6-12 for F1 protease with optimum activity at pH 9. This showed the SpSKF4 protease was more alkaline than the F1 protease from *G. stearothermophilus* F1. The stability of protease enzymes within the pH range of 8.5 to 10 is regarded as favorable for detergent formulation in the detergent industry [36]. A recombinant serine protease from *Bacillus cereus* was produced, according to Suberu, *et al*. [31], and it showed activity between the pH ranges of 6 and 11, with pH 10 showing the best protease activity. Like this, Lakshmi *et al*. [66] discovered that the purified thermostable alkaline protease isolated from *Bacillus cereus* strain S8 had great stability within the pH range of 7 to 12 and displayed outstanding activity with casein at pH 10.0 [66]. These findings are in line with the observations made in the present study. Generally, the pH optimum for alkaline proteases is between the pH range of 9 and 11 [72-74]. However, there are some exceptions with a higher optimum pH of 11.5 recorded [3, 75, 76], as well as a pH of 11-12 [76-78] and even pH 12–13 [76, 79, 80]. Alkaline proteases are usually stable at pH 6-12 [72, 81]. Most commercially important subtilisin-like proteases are active at a pH range of 8-12 [1, 76]. The high stability of the SpSKF4 protease enzyme makes it a good enzyme for detergent formulation and other industrial applications.

### Effects of Metal Ions on the Protease Activity

**Fig. 7:**
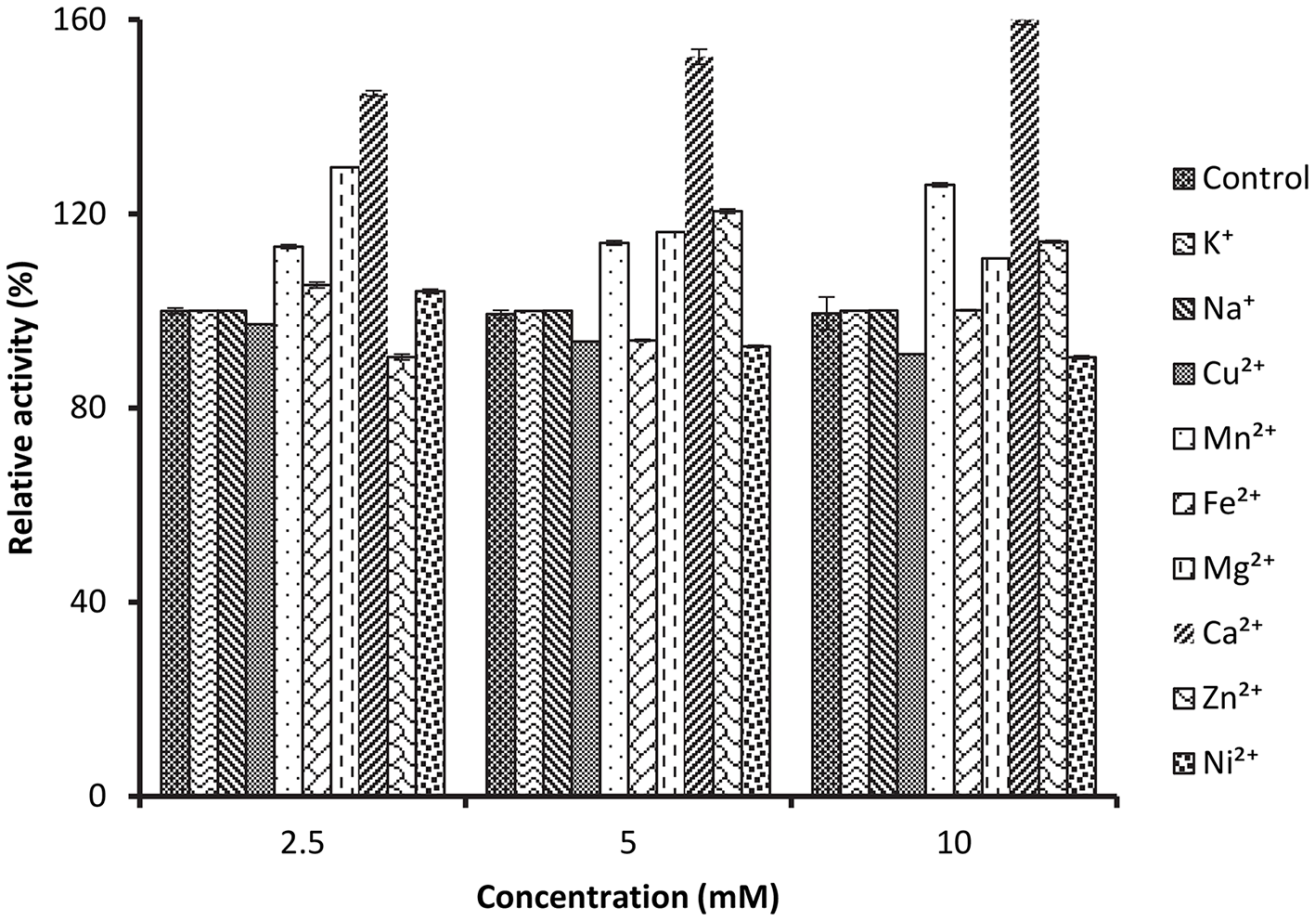
Effect of metal ions on activity of SpSKF4 protease.

The influence of metal ions was carried out with the incubation of the enzyme in pH 10 buffer at the optimum temperature of 80°C in various concentrations of 2.5, 5 and 10 mM (Fig. 7). The studys showed that Fe^2+^, Mg^2+^, Ca^2+^, Mn^2+^ and Ni^2+^ increased the activity of SpSKF4 protease at different concentrations. The findings showed that the enzyme was active in all the metal ions. The activity was increased by about 60% by divalent metals such as Ca^2+^, Zn^2+^; however, Cu^2+^ reduces the activity of the enzyme (Fig. 7). In previous studies, [36] reported the enhancement of the activity of protease from A. pallidius by Ca^2+^ and Mg^2+^, and Mn^2+^. Researchers [64] reported increased protease activity of R-H protease from *Geobacillus* sp. YMTC 1049 in the presence of Ca^2+^ and Mg^2+^, by 55 and 45%, respectively. The results also agree with [63] in which Mn^2+^ and Ca^2+^ ions increased the thermostability and activity of the protease from *B. stearothermophilus* F1. The addition of Mg^2+^ and Ca^2+^ increased the enzyme activity of protease from *B. licheniformis* MP1 by about 13% and 15%, respectively [11, 83]. This agrees with the present research, though Mg^2+^ and Ca^2+^ increase the activity of SpSKF4 protease better than the previous reported work. The observed elevation in enzyme activity in the presence of Ca^2+^ can be attributed to the stabilization of the enzyme in its active conformation, rather than the ion's direct involvement in the catalytic reaction [82]. Metal cations such as Ca^2+^ and Mg^2+^ generally influence the activity and stability of protease enzymes at different amounts [82]. Thermal stability and alkaline protease activity are increased at higher temperatures when adding Ca^2+^ [86-89]. Many enzymes need calcium ions as inducers and stabilizers by protecting them from conformational alterations [74]. Metal ions such as, Ca^2+^, Mg^2+^, and Mn^2+^ have been reported to positively regulate activity of alkaline protease from *B. circulans* [74, 85]. Actually, metal ions such as calcium give proteins structural and thermal stability. They also play a crucial part in preventing the denaturation of the enzyme brought on by high temperatures by assisting in the maintenance of the enzyme's active conformation at such temperatures [90-93].

### Effect of Organic Solvents on Protease Activity

The investigation on the impact of organic solvents was conducted at the enzyme's optimal temperature and at a pH of 10. Various organic solvents, including ethanol, methanol, isopropanol, butanol, acetone, chloroform, and n-hexane were employed at different concentrations (10, 30, and 50%). This test was conducted without any metal ions and was carried out over a period of 1 h.

**Fig. 8:**
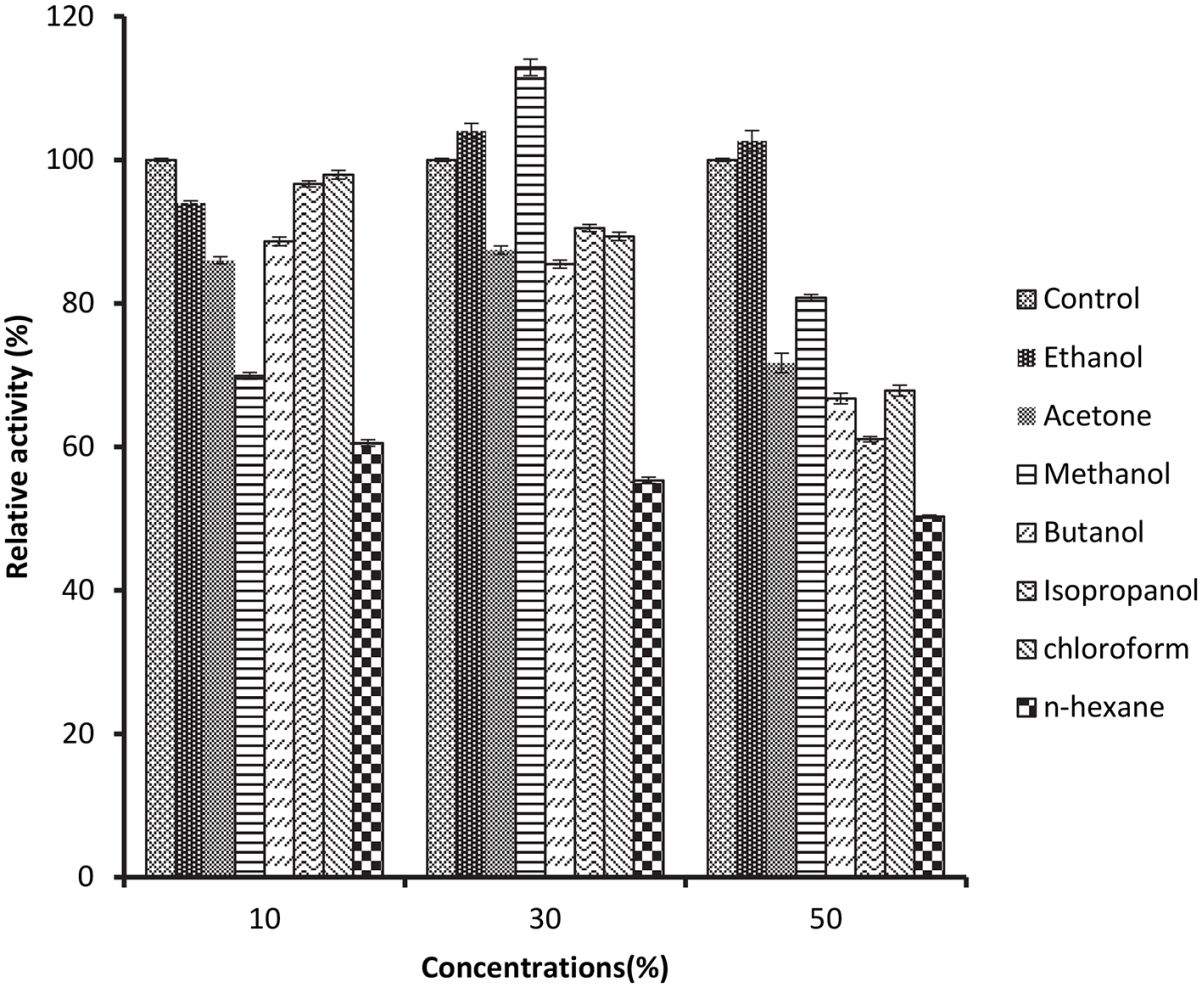
effect of organic solvents on activity protease.

The results of the relative activity and stability in organic solvents are showed in (Fig. 8). The control experiment is indicated by the enzyme activity without the addition of organic solvent and was taken as 100%. The results indicated the enzyme displayed considerable strength in the presence of most of the organic solvents. However, the enzyme's activity was reduced by half at a 50% concentration of n-hexane. In the presence of other organic solvents such as isopropanol, butanol, acetone, and chloroform, the enzyme maintained more than 60% of its activity at all tested concentrations. Interestingly, ethanol enhanced the enzyme's activity by approximately 4% at a 30% concentration and by approximately 2% at a 50% concentration (Fig. 8). Additionally, methanol increased the enzyme's activity by around 10% at a 30% concentration (Fig. 8). These findings provide valuable insights into the enzyme's behavior in the presence of various organic solvents, which can be crucial in industrial applications and process optimization.

The effect of various organic solvents on the activity of protease has been reported by previous researchers who examined the influence of water-soluble organic solvents such as ethanol, diethyl ether, methanol, and hexane on the stability of purified enzyme from *Bacillus licheniformis* MP1 at a concentration of 25% (v/v) [83]. The enzyme's half-life was found to increase to 56, 49, and 44 h, respectively, compared to the absence of organic solvent at 20 h. Isopropanol, however reduced the stability of *B. licheniformis* MP1 [83]. The results of other researchers who used similar organic solvents agreed with our findings [93-98]. However, SpSKF4 protease shows higher stability than most of the previous studies. It has been emphasized that the natural protease's inherent ability to maintain its activity and stability within organic solvents without requiring any alterations is an essential quality in a variety of application processes [74, 99]. Particularly, the SpSKF4 protease exhibits a remarkable characteristic with its excellent stability when exposed to organic solvents. This property is of significant interest and relevance in diverse industrial applications where the enzyme's performance within organic solvents is critical for the efficiency and effectiveness of various processes.

Due to the exceptional features of its stability in very many organic solvents, the SpSKF4 alkaline protease will be of great importance in industrial as well as many biotechnological applications.

### Effect of Surfactant on Protease Activity

**Fig. 9:**
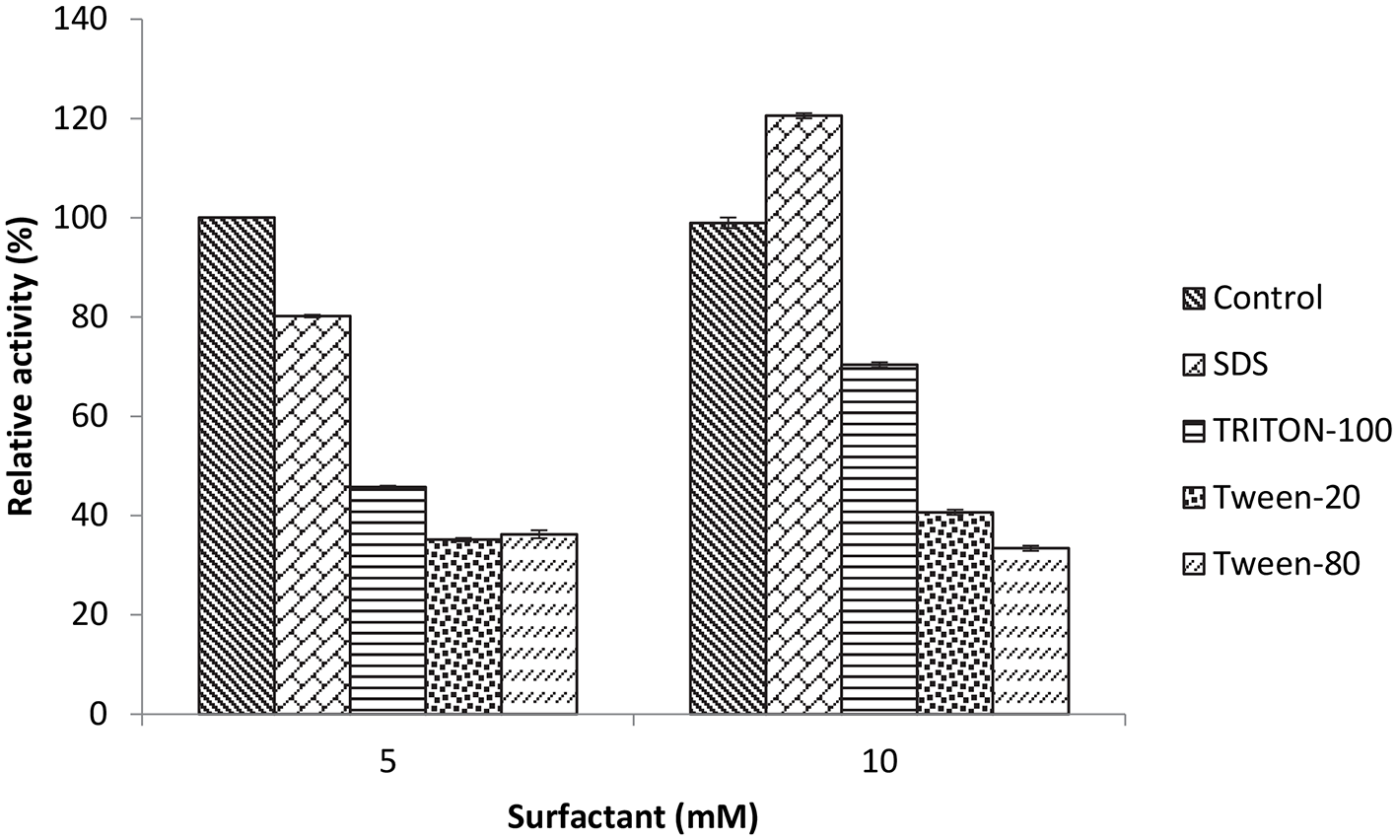
Effect of surfactant on the activity of partially purified SpSKF4 protease.

The influence of surfactant on the activity of the SpSKF4 enzyme was performed at the optimum pH 10 with different concentrations at 5 and 10 mM. The surfactants studied include SDS, Triton-100, Tween-100, Tween 20, and Tween-80. The findings revealed that the protease enzyme, which was cloned from *G. thermoglucosidasius* SKF4, maintained its stability to a significant degree in only a limited number of the surfactants tested (Fig. 9).

The protease showed weak stability with Tween-20 and Tween-80 at 10 mM, with 40 and 33% stability, respectively. However, SDS increased the enzyme's activity and stability by 20%, and at a concentration of 10 mM with Triton-100, the protease preserved 70% of its activity. The enzyme's stability against Triton X100 was observed to be 73% after 1 h of treatment with a maximum amount of surfactants of 5% at 30°C. The enzyme retained about 40% of its activity in the surfactants. However, the enzyme activity was increased by 20% of SDS (Fig. 9). Various studies on the effect of surfactants have been reported. The recombinant thermostable protease Tcsp demonstrates important residual activity even after pre-incubation with surfactants, such as SDS, under high-temperature conditions [13, 100]. This agrees with the result obtained for SpSKF4 in the present study which has over 70 % stability in Triton X100. Some protease enzymes in previous studies are shown to be stable in the presence of SDS, but the numbers are few [36]. The SpSKF4 protease showed high stability in SDS. The fact that the enzyme was still active (40-60 %) with all the surfactants tested and showed high stability with SDS indicates the protease enzyme is valuable as a potential industrial and biotechnological enzyme, especially as a detergent additive. The stability of SpSKF4 protease in the presence of denaturants like SDS suggests that its protein possesses a tightly packed structure with a highly rigid native conformation [101]. There is a relationship between the stability of a protein and the structural property of the protein. A well-packed protein will naturally result in increased thermostability which has a direct correlation with its rigidity [102, 103]. The surfactant and chelators may affect the protein’s native conformation differently, thus increasing the flexibility of its conformation.

For a protease enzyme to be used in detergent additives and the laundry industry, its stability and activity in alkaline pH, high temperatures, as well as its ability to withstand detergent agents such as surfactants, bleaching agents, bleach activators, fabric softeners, and other formulations should be guaranteed [104]. The stability of SpSKF4 protease in SDS and other surfactants shows the potential for its application in the detergent industry [04]. The capacity of SpSKF4 protease to remain stable in the surfactants clearly demonstrates its high thermostability.

### Effect of Commercial Detergent on Protease Activity

**Fig. 10:**
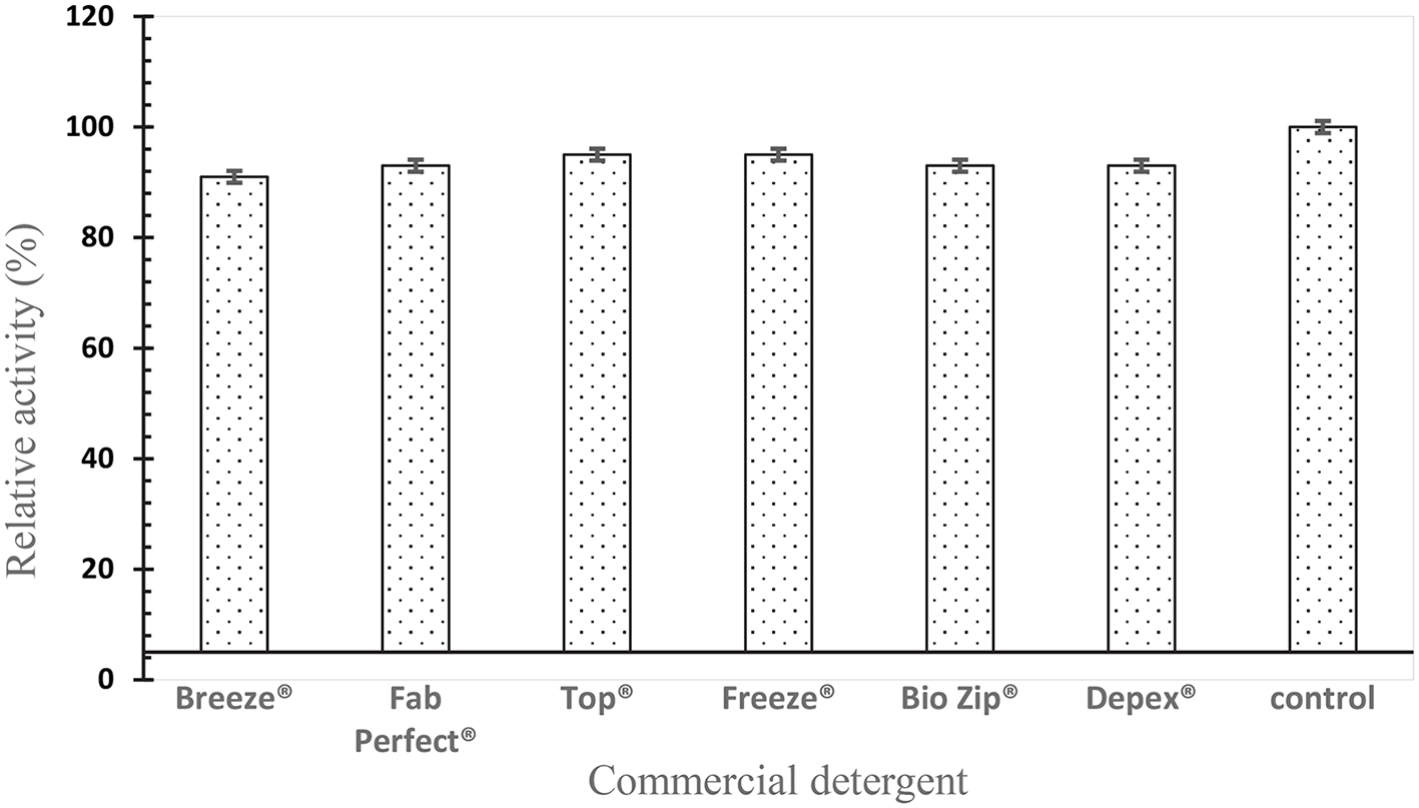
SpSKF4 protease compatibility with commercial detergents. The result showed average of 91% compatibility. The control test is the activity of SpSKF4 protease only without detergent.

Enzymes must function efficiently and have a greater laundry activity during the removal of substrates in the cleaning process by enzyme-based detergent formulation. Therefore, stability of recombinant SpSKF4 protease in the presence of some trademarked commercial detergents was investigated. Fig. 10 shows the compatibility of the enzyme with some commercially available detergents. The enzyme retained an average of over 90% of its activity after treatment with all the commercial detergents used (Fig. 10). Several alkaline proteases' stability in common detergents has been thoroughly investigated by numerous researchers. The recombinant alkaline protease was isolated from *G. thermoglucosidasius* SKF4, however, this study is the first to describe its detergent stability. Researchers from related studies have also looked at the stability of proteases in various commercial detergent formulations. [28, 105-107].

The findings of previous studies agree with the present investigation. However, SpSKSF4 protease shows better stability and compatibility in many detergents investigated. The compatibility studies of thermostable protease from *G. toebii* LBT 77 and *B. subtilis* RD7 [11, 31], with some commercial detergents with residual activity of over 90%, agreed with the results our findings. The report of [108] indicates the high activity of protease from *Bacillus*, *Alcaligene*, and *Pseudomonas* with different commercial detergents, such as Surf, Wheel, and Ariel, but very poor activity and compatibility with Patanjali. Researchers also reported that 70% of residual activity of an alkaline protease was retained with some commercial detergents [109]. This is however lower than the results produced from SpSKF4 protease. The alkaline SpSKF4 protease’s compatibility in all the commercial detergents is a confirmation of its possible use in detergent formulations, which is a result of its high stability and activities in organic solvent and surfactants. According to existing literature, a quality detergent’s enzymes are expected to retain their activity in the presence of laundry detergents during the washing process.

### Effect of Inhibitors on Protease Activity

**Fig. 11:**
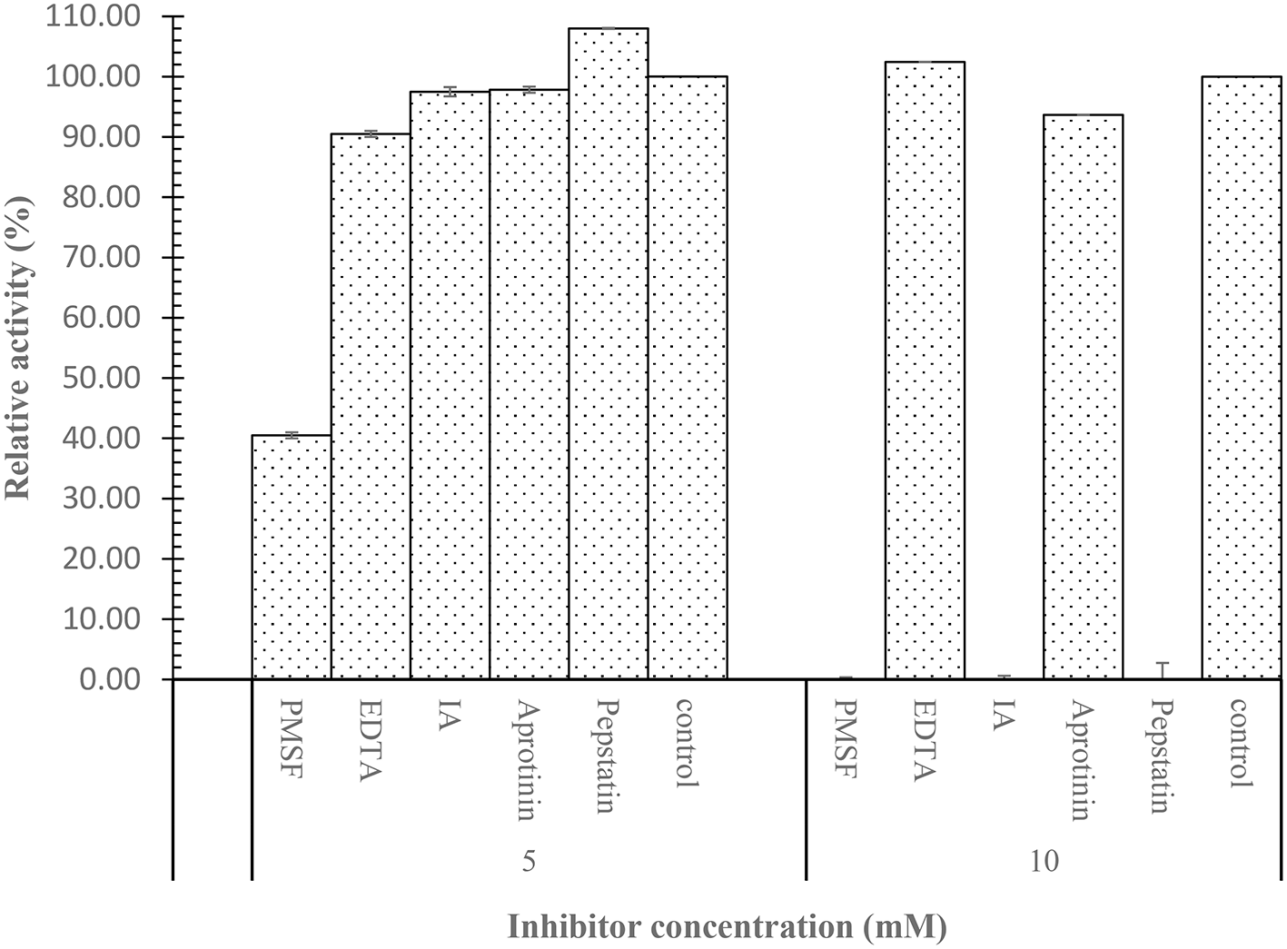
Effect of different inhibitors on activity and stability of SpSKF4 protease with total inactivation of the enzyme Phenylmethylsufonyl fluoride (PMSF) at 10 mM concentration. Indoacetimide (IA), Ethylene diamine tetraacetic acid (EDTA).

The influence of various inhibitors was assessed by incubating the enzyme in different concentrations of the inhibitors. The findings, as shown by Fig. 11, indicated that the activity of SpSKF4 protease was totally suppressed by PMSF (phenylmethylsulfonyl fluoride), a well-known serine protease inhibitor, at a concentration of 10 mM. Particularly, the complete inhibition of the enzyme by PMSF suggests that SpSKF4 is a serine protease.

Other inhibitors, such as EDTA (metalloprotease inhibitor), aprotinin, indoacetimide, and pepstatin (an aspartic protease inhibitor), did not inhibit the protease enzyme. EDTA actually increased the activity by 2% at a concentration of 10 mM, which shows the SpSKF4 is not a metalloprotease, while pepstatin increased the activity by 8% at concentration of 5% (Fig. 11). The result indicated that SpSKF4 protease may be resistant to EDTA metal chelator. This finding emphasizes the SpSKF4 protease's classification as a serine protease by indicating that the serine residue(s) is/are critical for the catalytic activity. Particularly for possible uses in detergent formulations, the SpSKF4 protease's sensitivity to chelators is a useful characteristic [114]. The capacity of chelating agents to act as both a water softener and a stain remover makes them a prominent ingredient in detergents [114].

Numerous researchers have looked into how inhibitors affect protease activity. Similar findings were reported by Mechri *et al*. [110], who found that the serine protease inhibitors PMSF and DIFP, both of which are well-known inhibitors of serine proteases, dramatically reduced the activity of a thermostable protease from *Aeribacillus pallidus* strain VP3. It is widely known that many serine proteases become less active when they are inhibited by substances like DFP (diisopropyl fluorophosphate) or PMSF (phenylmethylsulfonyl fluoride). A total loss of serine protease activity is known to occur when PMSF, in particular, binds directly to the active site serine residue in serine hydrolases [74, 86, 114, 115]. Significantly, PMSF does not bind to any additional serine residues found within the protein [3]. Our findings showed that PMSF inhibited SpSKF4 activity when the concentration was raised to 10 mM, but that there was a drop in the activity at a lower concentration (Fig. 11), which shows SpSKF4 to be a serine protease. As a chelating agent EDTA has been reported to inhibit protease [110]; however, SpSKF4 activity was increased by EDTA and pepstatin by 2 and 8%, respectively. Other researchers have reported increase in activity by chelators such as EDTA and EGTA [63, 64, 112, 113]. The report of Xu *et al*. [111] also indicated PMSF inhibits a recombinant alkaline protease from *B. subtilis*-D2, but the enzyme is unaffected by EDTA. This also agrees with the findings of the present study. Since EDTA increased the activity of SpSKF4 protease, this indicates the enzyme does not belong to the metalloprotease group or aspartic protease. Pepstatin, an aspartic protease inhibitor, increased the protease activity, showing that SpSKF4 protease is not aspartic protease.

### Effect of Oxidizing and Reducing Agents on Protease Activity

**Fig. 12:**
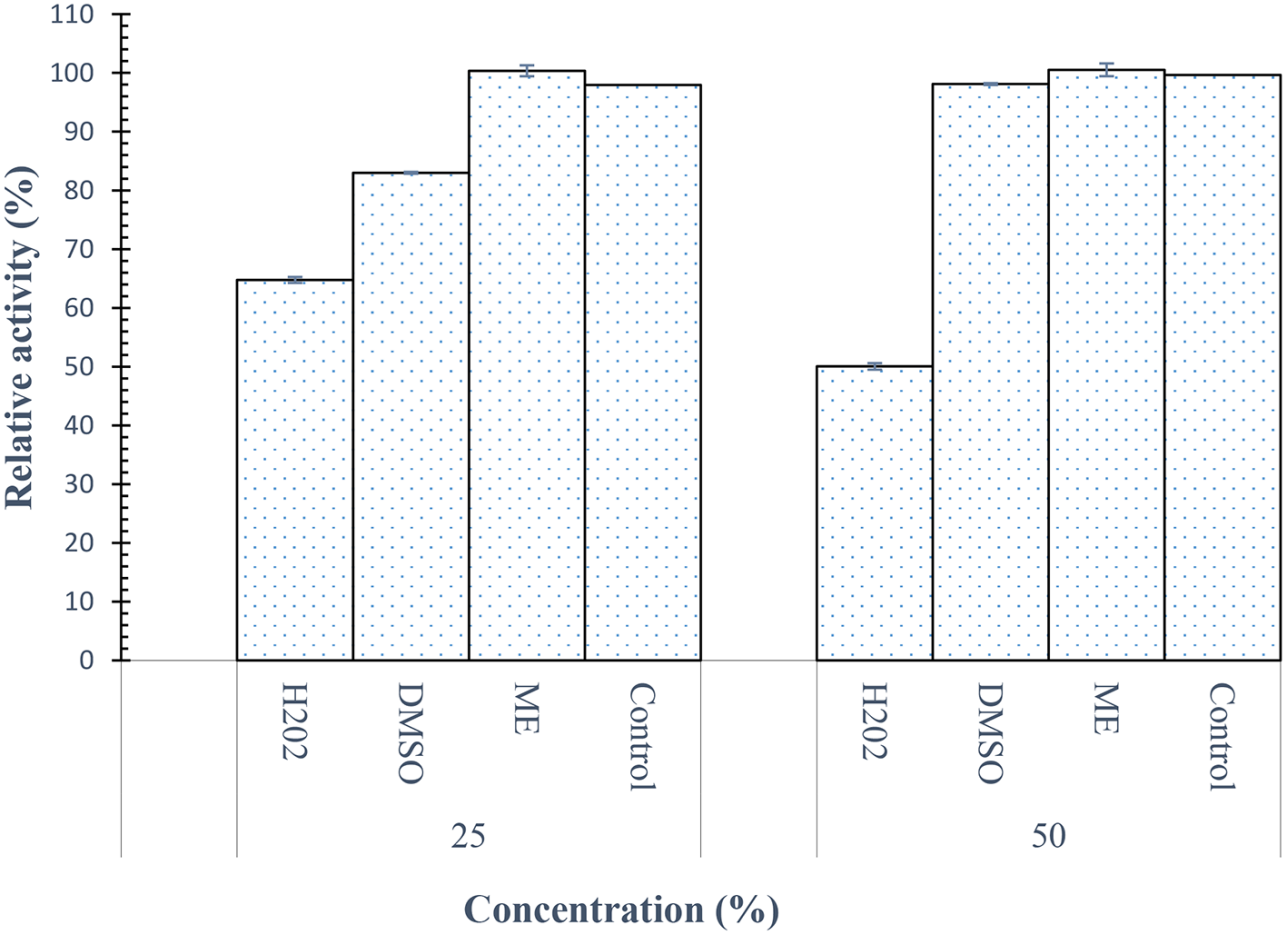
Effect of reducing and oxidizing agents on the activity and stability of SpSKF4 protease at optimum temperature of 80°C. ME (β-Mercaptoethanol).

The effect of oxidizing and reducing agents such as dimethyl sulfoxide (DMSO), H_2_O_2_ and β-Mercaptoethanol (ME) was tested at different concentrations on the enzyme. The alkaline protease SpSKF4 shows remarkable stability in both oxidizing and reducing agents. Fig. 12 shows the influence of both reducing and oxidizing agents on the activity of the protease enzyme. The results revealed that the enzyme preserved approximately 60% of its activity in the presence of the oxidizing agents, while it remained unaffected by the reducing agents, such as β-mercaptoethanol. There have been several reports describing the effects of dimethyl sulfoxide (DMSO) and hydrogen peroxide (H2O2) on the activity of proteases, indicating that these agents can significantly influence the behavior and stability of these enzymes under varying conditions.

The activity of protease from *Aspergillus oryzae* CH93 was reduced by 30% by DMSO and also reduced by H_2_O_2_ (Salihi *et al*.) [116]. This result agrees with our findings, although SpSKF4 retained 98% of its activity in 50%concentration of DMSO while 65% of its activity was retained at 25% H_2_O_2_ (Fig. 12). This demonstrates that SpSKF4 is more active and stable than other previous findings. The results obtained for the SpSKF4 protease are consistent with those reported by Suberu *et al*. [31] on the impact of β-mercaptoethanol on the activity of the recombinant serine protease from *B. subtilis* RD7. It is evident how stable the SpSKF4 protease is in the presence of reducing agents. This can observed from the results, where the enzyme is unaffected by b-mercaptoethanol. The SpSKF4 protease enzyme's stability shown in solutions with both oxidizing and reducing chemicals demonstrated its significance for a variety of industrial and biotechnological situations, underscoring its potential versatility and utility.

### Substrate Specificity of the Protease

Different substrate at 1% concentration were tested on the SpSKF4 protease.

According to the data presented in Table 2, we observed that casein serves as the most effective substrate for protease activity, with the enzyme exhibiting its highest activity at 353 U/ml, representing 100% activity. However, the ability of the enzyme to hydrolyze various proteins, such as azocasein, BSA, gelatin, keratin, and others (Table 2), represents an essential feature of alkaline protease [117, 118]. Similar results that showed high specificity for casein have been reported, such as the highest activity for casein with F1 protease from *G. stearothermophilus* F1 [63]. Yildirim *et al*. [36] also reported the highest substrate activity (100%) for protease enzyme from *A. pallidus* C10 using casein substrate. These findings agree with the present study. Other alkaline proteases from *B. circulans* MTCC [47] and *B. pumilus* MCAS8 [119] have also shown the greatest substrate activity against casein. Sari *et al*.[120] reported similar activity of protease from *B. circulans* M34, which has activity toward casein, oval albumin, and BSA, but not with keratin and haemoglobin. Proteases from *Bacillus halodurans* S373 CAS6 [106] and *B. subtilis* DR8806 [121] were observed to be active against casein compared to other substrates. All these findings agree with the present study. The alkaline SpSKF4 protease, however, demonstrated the highest activity toward casein because protease enzyme is highly substrate-specific [122]. This can be related to the enzyme's ability to maximize the substrate's binding energy, which is used to determine substrate affinity.

### Applications of Serine Protease

**Washing Capacity of SpSKF4 Protease to Remove Blood Stains**

**Fig. 13:**
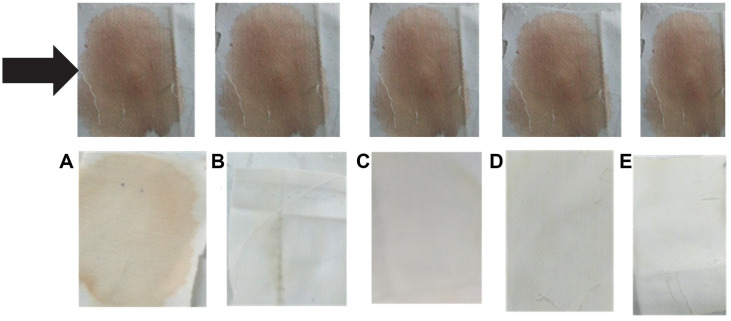
Removal of blood from white cloth pieces stained with blood, Black arrow: sun dried (12 h) blood stained cloth before removal, (A) blood stained cloth after incubation with water, (B) 1% detergent (v/v) solution incubated in water at 70°C to inactivate enzyme, (C) partially purified alkaline protease *B. licheniformis* 2D55 prepared in 1% detergent solution (positive control), (D) partially purified SpSKF4 (352 U) alkaline protease only, (E) partially purified SpSKF4 alkaline protease prepared in 1% (w/v) detergent.

The SpSKF4 protease was tested on white pieces of blood-stained cloth to see whether it may improve washing performance when added to detergent (Fig. 13). The enzyme's ability to efficiently break down and remove protein-based stains, like those from blood, was probably the main focus of this test, which was intended to evaluate its potential value as an active ingredient in detergent formulations for better stain removal and cleaning effectiveness. The investigation was conducted within 10-45 min in three replicates, and there was no significant difference in the visual observations of each replicate of each experiment. The effectiveness of the serine alkaline protease (350 U/ml) in removing protein stains, specifically blood stains, was evaluated at a temperature of 80°C and in the presence of a 1% (v/v) detergent solution.

When applied separately, the detergent (after its enzyme has been inactivated by heating at 70°C for 10 min) was unable to remove a little amount of discoloration (blood stain) after 10 min, but partially removed the stain after 45 min. Less of the stain was removed when water was substituted with detergent and without enzyme. However, the stains were greatly removed in 10 min and completely removed after 45 min at 80°C with 350 U/ml SpSKF4 protease enzyme and 1% (v/v) detergent. However, when only the SpSKF4 protease was used and without detergent, the blood stain was removed partially after 45 min (Fig. 13).

Serine protease SpSKF4 was successfully used to remove blood stains from cloth when combined with detergent (Fig. 13). Various proteases derived from different bacterial sources have been used as detergent additives. Patil *et al*. [47] described the use of protease from *B. circulans* MTCC 7942 for removing blood stains, which required a time period of 1 h. In contrast, the SpSKF4 protease demonstrated faster stain removal with time. Additionally, proteases from various other *Bacillus* species, such as *B. subtilis* PE-11 [118], *B. circulans* [85], *B. mojavensis* A 21 [13], and *B. alveayuensis* CAS 5 [106], have also been utilized as detergent additives. These proteases play a crucial role in enhancing the performance of detergents by assisting in the breakdown and removal of protein-based stains, and the choice of protease may depend on factors like efficiency, stability, and compatibility with detergent formulations.

Marathe *et al*. [108] also reported the use of proteases from *Bacillus* sp., *Alcaligenes faecalis* and *Pseudomonas aeruginosa* for the removal of bloodstain from cloth materials. These previous results agree with the present study; however, the enzyme SpSKF4 protease showed better efficiency in the time of stains removal and activity. These results show the likelihood of the efficiency of the SpSKF4 protease for potential application as a cleaning additive agent in commercial detergent formulation. To improve the ability of detergents to remove stains, serine alkaline proteases have been added as biobuilders [31, 123, 124]. These enzymes are essential for hydrolyzing and removing proteinaceous elements from discolored clothing. Their activity extends beyond obvious stains like blood (Fig. 13) and includes less obvious remnants from bodily fluids, skin fragments, and other foods like egg yolk, fish, meat, and milk [31].

Proteinaceous residues tend to solidify on cloth during washing processes in the absence of proteases [31]. While bleaching compounds operate to break down undissolved dyes, high temperatures, an alkaline pH, and the action of surfactants and sequestering agents used in washing operations help to dissolve or disperse the majority of the dirt components [125]. However, proteinaceous substances often precipitate on the fabric during these processes. Failure to effectively remove these proteinaceous contaminants can lead to the development of a dull, grayish appearance on the fabric, imparting an overall unclean and unsightly look, especially after multiple wash cycles [125].

The SpSKF4 protease demonstrated stability and activity at high temperatures and metal ion concentrations, suggesting that it could be a useful biochemical for biotechnological applications. It could also be important in fields such as environmental bioremediation, the leather industry, and laundry as a detergent additive.

### Gelatinolysis of X-Ray Photographic Film for Recovery of Silver

To recover silver from X-ray photographic film, the SpSKF4 protease's gelatinolytic capacity was tested over the course of 3 h. This procedure was carried out at the enzyme's preferred conditions, especially at 80°C and pH 10. There were no observable differences in the experimental and biological replicate of each experiment. The efficiency of the SpSKF4 protease enzyme was assessed using a positive control using protease from *B. licheniformis* 2D55 and a negative control utilizing Glycine-NaOH buffer. We calculated how much protein was removed from the X-ray film after 1–3 h of hydrolysis. The results showed high activity of recombinant alkaline thermostable SpSKF4 protease against gelatin, which is a component of X-ray photographic film. During the experiment, we discovered that the protease effectively degraded the gelatin layer contained in the X-ray film within a relatively short period of time (60 min). This technique was carried out under ideal circumstances of 80°C and pH 10. The decline in protein concentration after 3 h of gelatinolysation (Table 3) indicates that an additional 2 h of hydrolysis led to a higher degree of gelatin degradation. The results revealed that after 1 h of X-ray film hydrolysis with SpSKF4 protease, the amount of protein generated was 0.35 mg/ml, and the protein concentration was significantly reduced to about 0.19 g/ml after 3 h (Table 3). The results further confirmed that SpSKF4 protease performed greater gelatinolysation and silver recovery than both *B. licheniformis* 2D55 protease and Glycine-NaOH buffer (Table 3).

**Fig. 14:**
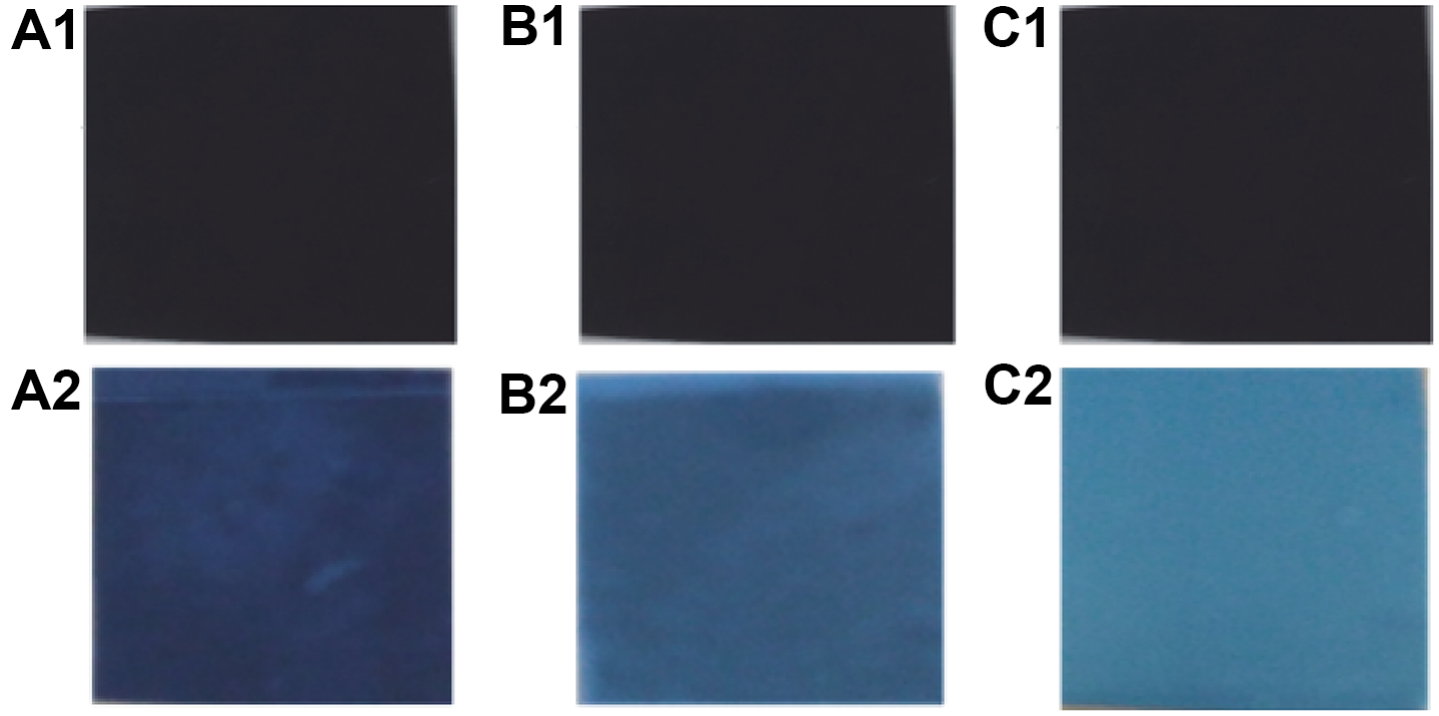
X-ray photographic film gelatinolysis for silver recovery using SpSKF4 protease from *G. thermoglucosidasius* SKF4: Letter A1, B1 and C1 shows X-ray film before exposure to enzyme treatment, A2 after exposure to partially purified *B. licheniformis* 2D55 protease for 3 h (positive control), B2 after treatment with Glycine-NaOH buffer only (Negative control), and C2 after exposure to SpSKF4 protease enzyme for 3 h. The experiment were performed in triplicates. There was no observable differences in the replicate for each experiment.

The results showed SpSKF4 protease has broad substrate specificity. The X-ray film treated with SpSKF4 protease appears lighter in color than other treatments, indicating that the SpSKF4 enzyme has a higher capacity for gelatin hydrolysis and X-ray stripping, resulting in greater silver recovery (Fig. 14). The experiment was performed in triplicate. There were no observable differences in the replicate for each experiment.

According to earlier studies [126], proteases are renowned for their significant gelatinolytic activity, which helps to make it easier to successfully recover silver from X-ray images. Enzymatic hydrolysis of photographic X-ray waste has been confirmed as a more advantageous method for silver recovery by researchers [47, 127]. Protease from *Purpureocillium lilacinum* has also been reported to show good gelatin hydrolyzing ability [128]. Singh and Bajaj [30] utilized a thermostable alkaline protease derived from *B. licheniformis* K-3 to achieve complete de-gelatinization of X-ray photographic film in a relatively short period of 20 min. The successful recovery of silver from X-ray photographic films using alkaline proteases produced by *B. subtilis*, *Streptomyces avermectnus*, and *Conidiobolus coronatus* has also been reported [126]. These findings agree with the results of the present research. The exceptional performance of the SpSKF4 protease in various biochemical reactions, coupled with its potential for recyclability, indicates its significant commercial value. Particularly, the stability of SpSKF4 protease at high temperature, its pH stability, and its potential applications such as in the removal of silver from used X-ray films, demonstrate the positive impact of the enzyme on both human activities and the environment.

## Figures and Tables

**Table 1:** Purification table of partial purified recombinant SpSKF4 protease.

Fraction	Volume (ml)	Total activity (U)	Total protein (mg)	Specific activity (U/mg)	Purification fold	Recovery (%)
Crude	25	3250	197	16.4	1	100
Heat treatment	25	2015	30.6	26.5	1.6	62
IMAC	16	352	2.5	141	8.6	11

**Table 2:** Specificity SpSKF4 Protease with Different Substrates.

Substrate	Protease activity ( U/ml)	SD
Bovine Serum Albumin (BSA)	234	±1.33
Casein	353	±1.31
Keratin	82	±1.17
Azocasein	251	±0.94
Haemoglobin	203.5	±0.62
Oval Albumin	254	±0.77
Gelatin	244	±0.85

**Table 3:** Amount of protein (mg/ml) generated during X-ray film gelatinolysis using partially purified SpSKF4 protease at 80°C and pH 10, *B. licheniformis* 2D55 at 45°C and pH 9 (positive control) and Glycine-NaOH buffer (negative control).

Time of hydrolysis in h	Protein (mg/ml)		
	SpSKF4 protease	*B.licheniformis* 2D55(control)	Glycine-NaOH buffer (control)
1	0.35 ± 0.05	0.65 ± 0.1	0.47 ± 0.13
2	0.26 ± 0.1	0.60 ± 0.12	0.30 ± 0.11
3	0.19 ± 0.08	0.45 ± 0.11	0.23 ± 0.09
